# Taking SCFAs produced by *Lactobacillus reuteri* orally reshapes gut microbiota and elicits antitumor responses

**DOI:** 10.1186/s12951-024-02506-4

**Published:** 2024-05-12

**Authors:** Nannan Li, Lili Niu, Yao Liu, Yang Wang, Xiaomin Su, Ce Xu, Zanya Sun, Huishu Guo, Jingru Gong, Shun Shen

**Affiliations:** 1 Central Laboratory, First Affiliated Hospital, Institute (College) of Integrative Medicine, Dalian Medical University, Dalian, 116021 China; 2 Pharmacy Department, Shanghai Pudong Hospital, Fudan University Pudong Medical Center, Shanghai, 201399 China; 3 Clinical Oncology Center, Shanghai Municipal Hospital of TCM, Shanghai University of Traditional Chinese Medicine, Shanghai, 200071 China

**Keywords:** Microgel, SCFAs, Intestinal flora, Butyrate, GPR109A

## Abstract

**Background:**

Colorectal cancer (CRC) incidence is increasing in recent years due to intestinal flora imbalance, making oral probiotics a hotspot for research. However, numerous studies related to intestinal flora regulation ignore its internal mechanisms without in-depth research.

**Results:**

Here, we developed a probiotic microgel delivery system (*L.r*@(SA-CS)_2_) through the layer-by-layer encapsulation technology of alginate (SA) and chitosan (CS) to improve gut microbiota dysbiosis and enhance anti-tumor therapeutic effect. Short chain fatty acids (SCFAs) produced by *L.r* have direct anti-tumor effects. Additionally, it reduces harmful bacteria such as *Proteobacteria and Fusobacteriota*, and through bacteria mutualophy increases beneficial bacteria such as *Bacteroidota and Firmicutes* which produce butyric acid. By binding to the G protein-coupled receptor 109A (GPR109A) on the surface of colonic epithelial cells, butyric acid can induce apoptosis in abnormal cells. Due to the low expression of GPR109A in colon cancer cells, MK-6892 (MK) can be used to stimulate GPR109A. With increased production of butyrate, activated GPR109A is able to bind more butyrate, which further promotes apoptosis of cancer cells and triggers an antitumor response.

**Conclusion:**

It appears that the oral administration of *L.r*@(SA-CS)_2_ microgels may provide a treatment option for CRC by modifying the gut microbiota.

**Supplementary Information:**

The online version contains supplementary material available at 10.1186/s12951-024-02506-4.

## Introduction

Colorectal cancer (CRC) is a multifactorial disease influenced by genetics, environment, and lifestyle, which ranks third in global mortality [1]. The human gut contains a large number of microorganisms. Typically, various gut flora maintain microbiological balance in the intestinal tract, providing nutrients to the body, defending against pathogen invasion, regulating the proliferation of intestinal mucosa, and participating in digestion, catabolism, and immunological functions [2, 3]. To some extent, intestinal flora determines health. An imbalance in the gut flora can affect the gut microenvironment and gut cell function [2–4]. For example, the number of beneficial bacteria decreases, which reduces the antioxidant capacity of the intestinal tract, aggravates the damage of the mucosal layer [5, 6], and some pathogenic bacteria can destroy pro-inflammatory and anti-inflammatory cytokines. Increased harmful bacteria can increase cancer cell proliferation, promote CRC progression, and limit the effectiveness of immunotherapy [6, 7]. Hence, decoding the mechanism linking the gut microbiota to CRC is therefore critical to treating CRC by modulating the gut microbiota. Oral probiotics have become increasingly popular in cancer treatment in recent years. Probiotics can work through a variety of different mechanisms [8–10]. As indirect nutrients produced by gut microbiota, SCFAs play an important physiological regulatory role [11–14]. Acetic acid, propionic acid, and butyric acid are the major components of SCFAs, which are essential for maintaining water and electrolyte balance, regulating immunity, and maintaining intestinal flora [14]. SCFAs can function in a variety of ways, with butyric acid having the most significant anti-tumor effects. We wondered if we could find a way to both regulate gut flora and increase the amount of SCFAs in the gut.

*Lactobacillus reuteri* (*L.r*), which has received a lot of attention in recent years, may help us with this problem. *L.r* is a Gram-positive facultative anaerobic bacterium of the genus *Lactobacillus*, which exists naturally in the intestines of almost all vertebrates and mammals [15]. In addition to colonizing the intestinal tract, *L.r* can also strengthen the physical barrier of the intestinal tract by reducing the transfer of microorganisms to extracellular tissues, and it is capable of producing antimicrobial molecules such as organic acids, ethanol, and retaining molecules that alter the composition of the gut microbiome and inhibit the colonization of pathogenic microorganisms. Through blood vessels and lymphoid tissues, *L.r* can reach tumors outside of the digestive tract and release indole-3-carbaldehyde, which stimulates killer T cells to attack tumors [16]. It acts as an anti-tumor agent primarily through its various metabolites and is a very valuable probiotic. Butyrate in SCFAs must bind to the GPR109A receptor for its antitumor effect. Under normal physiological conditions, the receptors bind to butyric acid produced by the metabolism of intestinal bacteria and induce apoptosis in abnormal cells. However, when colon cells become cancerous, the GPR109A receptor on their surface is underexpressed, resulting in a large amount of butyrate in the body that cannot fully bind to the receptor to function as an anti-tumor agent [17–19]. Therefore, it is not enough to simply increase the amount of butyric acid in the gut, and how to improve the expression of GPR109A is also a question that needs to be addressed. Currently, drugs developed for the GPR109A receptor are primarily agonists. Niacin drugs can stimulate this receptor, but these drugs have strong side effects. Several studies have shown that MK-6892 is a better agonist for GPR109A with fewer side effects. Studies are now focusing on the pharmacological effects of the combination. The application of MK to tumor models has not yet been discovered [20–22]. Perhaps we can make a bold attempt to explore a new approach to colon cancer based on oral probiotics to regulate the flora in combination with MK.

**Scheme 1:**
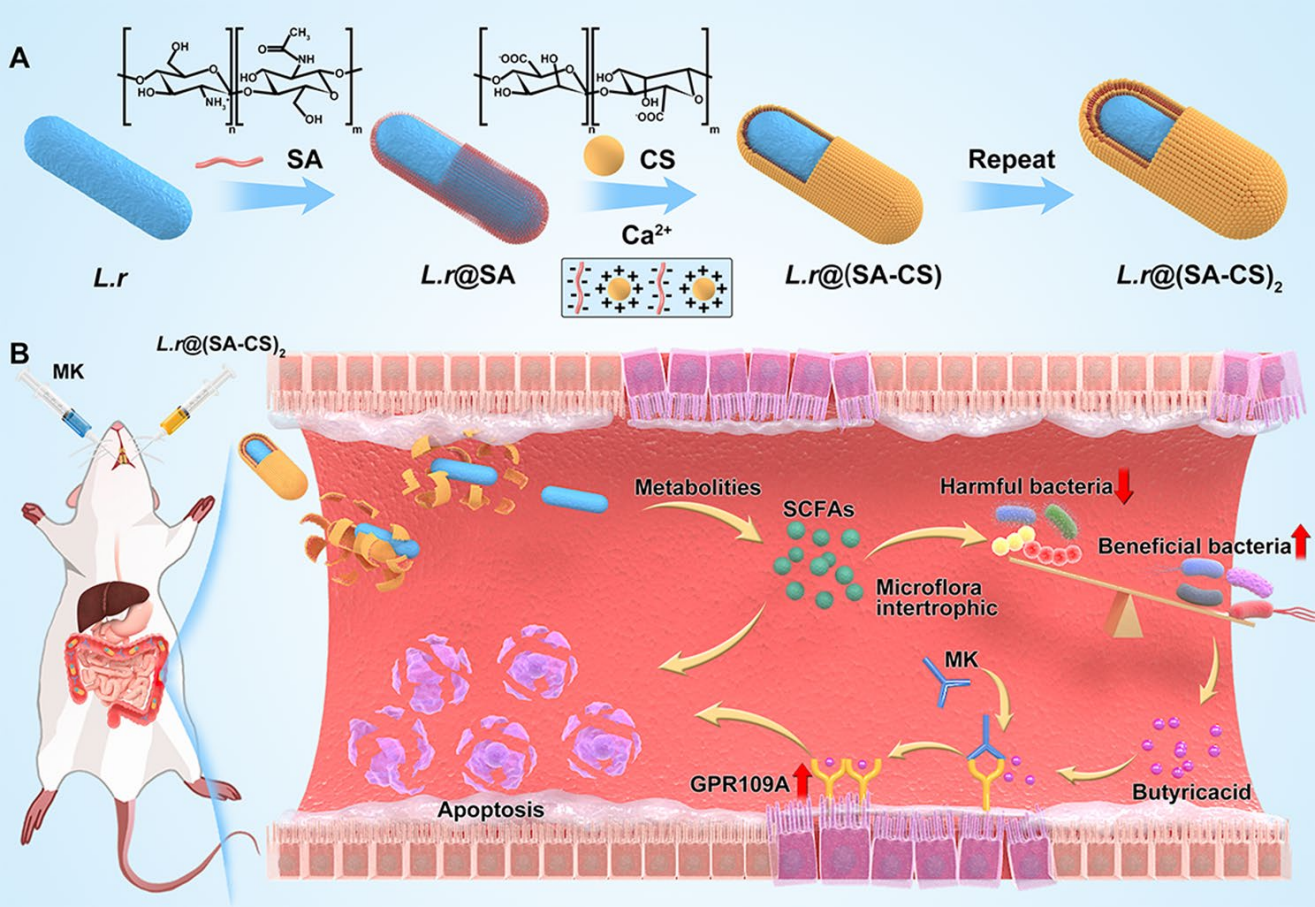
Schematic illustration of *L.r* @(SA-CS)_2_ microgel suppressed tumor growth by modulating intestinal flora. (**A**) Schematic illustration for the preparation of *L.r*@(SA-CS)_2_ by encapsulating *L.r* with SA and CS through the layer-by-layer strategy. (**B**) The tumor-bearing mice were intragastrically given *L.r*@(SA-CS)_2_ and then the MK. SA and CS enhanced both the resistance of probiotics against gastric insults and the delivery of living *L.r* into intestinal tract, which promoted tumor cells apoptosis and regulated the intestinal flora by producing SCFAs. Combined with MK, the microgel further enhanced anti-tumor effects.

Because probiotics are damaged by the harsh gastrointestinal environment during oral administration, which leads to reduced bacterial activity, there is an urgent need to develop oral delivery systems for probiotics to enhance probiotic colonisation in the gut [23, 24]. In this paper, a microgel multilayer encapsulation system *L.r*@(SA-CS)_2_ was constructed using the electrostatic interaction between SA and CS, layer by layer assembly and calcium chloride ion crosslinking, to protect *L.r* from gastric juice erosion successfully reached the intestinal tract (Scheme 1A). SA is the most commonly used natural bacterial encapsulation material [24–29], it readily forms gels when in contact with water, and the gels thus formed are PH sensitive and can expand and rupture in the gut to release bacteria [24, 30–33]. However, SA formed gels are porous and bulky, so they are usually combined with CS through electrostatic interactions to improve their stability and protection. After *L.r*@(SA-CS)_2_ protects *L.r* from the stomach juices and successfully reaches the intestines, SCFAs produced by *L.r* can cause apoptosis in some tumor cells, regulate intestinal flora, reduce harmful bacteria, and nourish other flora as nutrients, especially probiotics that produce butyric acid. Therefore, using the agonist MK to restore the expression of the GPR109A receptor on CRC cells, combined with the butyric acid produced by the metabolism of the flora, can further exert anti-tumor effects (Scheme 1B). The microgel in combination with MK showed high tumor inhibition in vivo and triggered an anti-tumor response with good biocompatibility.

## Experimental section

### Materials

Analytical grade chemicals were used without further purification. Sodium alginate, chitosan, calcium chloride, and sodium butyrate were purchased from Aladdin Reagent Co., Ltd. (Shanghai, China). MRS liquid and solid media were purchased from Haibo Biotech (Shanghai, China). Amplification of ampicillin, neomycin trisulfate hydrate, metronidazole, and vancomycin were purchased from Sigma-Aldrich. Mouse IFN-γ ELISA Kit, Mouse IL-6 ELISA Kit, Mouse TNF-α ELISA Kit, Annexin V-FITC/PI Apoptosis Detection Kit were all obtained from MultiSciences Biotch Co., Ltd., (Hangzhou, China). Mouse SCFA ELISA Kit was purchased from Zhucai Biotechnology Co., Ltd, (Shanghai, China). MK-6892 was obtained from MCE (USA). Cell Counting Kit-8 (CCK-8), DAPI, radioimmunoprecipitation assay (RIPA) lysis buffer, and BCA protein quantification kit were purchased from Beyotime Biotechnology Co. (Shanghai, China). GPR109A Rabbit mAb (A21974) was purchased from ABclonal (Wuhan, China). caspase-3 antibody #9662, Bcl-2 (D17C4) rabbit mAb #3498 and Bax (D2E11) rabbit mAb were purchased from Cell Signaling Technology (USA). Paraformaldehyde fixative, pepsin, and trypsin were purchased from Servicebio (Wuhan, China). Baclight LIVE/DEAD *L.r* activity assay kit from Thermo Fisher Scientific. D-Luciferin (sodium salt) was manufactured by Fushen Biotech (Shanghai, China). American Life Sciences (Gibco, Pittsburgh) provided the penicillin streptomycin solution, Roswell Park Memorial Institute 1640 medium, trypsin-ethylenediaminetetraacetic acid (EDTA, 0.05%), and fetal bovine serum.

CT26 cell was provided by Cancer Center, Fudan University and cultured in 1640 medium.

*L.r* Bio-53,258, was purchased from Beijing Biobowell Biotechnology Co., Ltd. After overnight culture on MRS liquid medium at 37 °C, *L.r* was diluted 1:100 into fresh MRS medium and incubated for 3 h, before being collected, centrifuged at 4750 rpm for 10 min, resuspended with pre-cooled PBS, diluted, and incubated overnight on MRS agar plates at 37 °C.

Male Balb/C mice, 6–8 weeks old, were purchased from Shanghai Shengchang Biotechnology Co., Ltd. (Shanghai, China), raised in the Medical Research and Innovation Center of Shanghai Pudong Hospital, and were fed for one week before the study using SPF Grade sterile mouse breeding feed and double distilled water, free access to water, and 12 h light cycles. To minimize pain, all operations were administered under isoflurane anesthesia. Shanghai Pudong Hospital’s Ethics Committee evaluated and approved all animal experiments (2022-KY-01).

### Preparation of *L.r*@(SA-CS)_2_ microgel

In order to determine the safe concentration of SA and CS alone on cells, different concentrations of SA (0, 1, 2, 5, 10, 15, 20 mg/mL) and CS (0, 1, 2, 4, 8, 16, 32 mg/mL) were co-cultured with 10^4^ CT26 cells for 24 h, and CCK-8 was used to measure the absorbance at 600 nm wavelength. The final concentrations of SA and CS used for encapsulation were 5 mg/mL and 1 mg/mL, respectively. Preparation of microgels involved mixing *L.r* with 5 mg/mL SA and stirring for 30 min, washing with PBS twice, 4750 rpm centrifugation for 5 min, resuspending with Saline (2 mL), then adding calcium chloride solution (1.1 mol/L) and stirring for 15 min, washing with PBS twice, 4750 rpm centrifugation for 5 min, and then resuspending with Saline (2 mL). In the end, the encapsulated cells were transferred to a CS solution with a concentration of 1 mg/mL and stirred for 30 min. The wrapping of the first layer was complete, the second and third layers were accumulated periodically, and so on.

### Characterization of *L.r*@(SA-CS)_2_ microgel

SEM (U8010, HITACHI) was used to characterize the micromorphology of *L.r*, *L.r*@(SA-CS), and *L.r*@(SA-CS)_2_. Detailed preparation of SEM samples was as follows: First, *L.r*, *L.r*@(SA-CS), and *L.r*@(SA-CS)_2_ were washed repeatedly, centrifuged, and fixed with electron microscope fixative at 4 °C for over 2 h. After blowing off the *L.r*, they were resuspended in the fixative, placed on a copper grid supported by carbon film, and adsorbed for five or ten minutes, the excess suspension being sucked up with filter paper. Following this, the copper grid with the sample adsorbed was rinsed three times with 0.1 mol/L PBS for 15 min each time, fixed with 1% osmic acid 0.1 mol/L PBS for 30 min, and rinsed three times with PBS (0.1 mol/L) for 15 min each time. After dehydration in ethanol water solution, the ethanol content was gradually increased from 50 to 100% for 15 min each. Lastly, added isoamyl acetate and allowed it to dry for 10–20 min. Once coated, observed the results. DLS (Nano ZS90, Malvern Instrument Co., Ltd.) was used to measure the particle size and Zeta potential of *L.r*, *L.r*@(SA-CS), and *L.r*@(SA-CS)_2_.

### Growth ability and activity of *L.r*@(SA-CS)_2_ microgel

Using one layer, two layers, and three layers of *L.r*, adjusted the samples of each group as needed to an optical density of approximately 1 × 10^7^ CFUs/mL in MRS medium (12 mL), 37 °C, 170 rpm shaker growth. At the predetermined time point, took 100 µL of *L.r*, *L.r*@(SA-CS), *L.r*@(SA-CS)_2_, and *L.r*@(SA-CS)_3_, then assessed their growth curve using a microplate reader at a wavelength of 600 nm. *L.r*’s viability detection with AlarmarBlue, 100 µL of the *L.r*’s solution should be taken from each group, and 10 µL of the detection solution should be poured into each well. The absorption at 570 nm was measured after 12 h incubation.

### Stability of *L.r*@(SA-CS)_2_ microgel in vitro and in vivo gastrointestinal fluids

In Vitro: *L.r* (4.0 × 10^5^ CFUs) were suspended in 1 mL of SGF (pH 1.2, containing 2 g sodium chloride, 3.2 g pepsin, 7 mL hydrochloric acid per 1 L water), and incubated at 37 °C with gentle shaking. At predetermined time points, *L.r* were diluted and coated on a solid agar plate (MRS), and incubated overnight at 37 °C after they were rinsed with PBS and extracted from the culture medium to count. The *L.r* (4.0 × 10^5^ CFUs) were cultured in 1 mL of SIF (pH 6.8, containing 0.2 M sodium hydroxide solution, 6.8 g potassium dihydrogen phosphate, 10 g trypsin per 1 L of water), shaking gently at 37 °C. For *L.r*’s enumeration, 50 mL droplets were collected at predetermine ed times, dispersed on MRS solid agar plates, and incubated overnight at 37 °C. Similarly, 4 × 10^5^ CFUs of *L.r* were resuspended in 1 mL of medium supplemented with bile salts (0.3 mg/mL) or ampicillin (10 mg/mL) for resistance testing.

In Vivo: upon gavage of *L.r*, *L.r*@(SA-CS) and *L.r*@(SA-CS)_2_ (4 × 10^8^ CFUs), mice were euthanized at specified time points, and the contents of the stomach, small intestine, cecum, and colon were collected. Dilution spreads were spread on MRS solid agar plates and incubated overnight at 37 °C for *L.r*’s counting.

### Cell viability assay

Cell viability was examined with CCK-8 assay. The CT26 cells should be inoculated into 96 well plates after the cultured cells are 80% full. The experimental groups *L.r* and *L.r*@(SA-CS)_2_ were shaken at a temperature of 37 °C for three hours in advance, and then the same amount of *L.r* were added to each well and incubated for 10 h at that temperature. After removing the culture medium 1640, washed the wells twice with PBS, added the CCK-8 reagent (1640: CCK-8 = 10:1), incubated for another 1.5 h, and measured the absorbance (OD value) at 450 nm with a Multidetection Microplate reader (BioTek, USA). Cell viability (%) = (sample-blank)/(control-blank) × 100%.

### Western blot assay

CT26 cells were spread evenly in a 6 well plate and cultured for 24 h. For the experimental groups, *L.r* and *L.r*@(SA-CS)_2_, the cells were shaken overnight at 37 °C in advance, and the supernatant was collected after centrifugation at 4750 rpm for 10 min. It was then added to each well with an equal amount of filtered supernatant, incubated for 10 h, washed three times with PBS, and the cells were collected and lysed. Cell lysates were centrifuged at 12,000 rpm for 15 min at 4 °C, and protein content was measured using a BCA protein kit after supernatant had been collected. Cell supernatant was processed according to Western Blot standard instructions. Using ImageJ software, we analyzed the experimental results semi-quantitatively.

The expression of apoptosis proteins in tumor tissues was assessed using western blot analysis. Following completion of the model, CT26 mice were subjected to in situ colon cancer and the treatment time point was recorded as day 0 (0d). The experimental groups were designated as follows: G1:Saline, G2:MK, G3:*L.r*, G4:*L.r*@(SA-CS)_2_; G5:*L.r* + MK, G6:*L.r*@(SA-CS)_2_+MK. Intragastric administration of bacteria (*L.r* or *L.r*@(SA-CS)_2_) was performed at 0d, 3d, 7d, and 11d, while intragastric administration of MK occurred at 4d, 6d, 8d, 10d, and 12d. Tumor tissue samples were collected at day 21 for further analysis. Equal amounts of tissue samples were weighed and sectioned before being homogenized with a tissue homogenizer in cooled PBS until no visible solid particles remained. After standing on ice for five minutes, the supernatant was carefully transferred into another pre-cooled clean centrifuge tube followed by centrifugation at 4℃ and 500 g for approximately two to three minutes. The resulting supernatant was discarded. To every twenty microliters of cell volume, add two microliters of protease inhibitor mixture along with pre-cooled Buffer A (200 µL), then vortex oscillate vigorously for fifteen seconds before placing on ice for ten minutes. Vortex oscillate again briefly before centrifuging at a speed of approximately16,000 g for five minutes at a temperature of -4 ℃ to obtain plasma protein by quickly aspirating the supernatant into another pre-cooled clean centrifuge tube. Total protein content was determined using a BCA protein kit following standard instructions when analyzing the collected cell supernatants. The collected cell supernatants were performed according to the standard instructions for Western Blot.

### Flow cytometric analysis

Plating CT26 cells at 3 × 10^5^ per well in a 6 well plate for 24 h. The experimental groups *L.r*, *L.r*@(SA-CS)_2_ were shaken at 37 °C for three hours in advance, then added the same amount of *L.r* to each well and incubated for ten hours at that temperature. The supernatant was collected, digested for 30 s with EDTA-free trypsin, and then the cells were collected, centrifuged together with the supernatant, washed twice with PBS, added Annexin V-FITC/PI dye, and incubated for 30 min at 37 °C. By using flow cytometry, the excess dye was subsequently washed off by washing with PBS. Live cells emit no fluorescence, early apoptotic cells emit green fluorescence (Annexin V) and dead/necrotic/late apoptotic cells emit both red (PI) and green fluorescence (Annexin V).

### Bacterial metabolites detection

Bacterial supernatant: the *L.r* (4.0 × 10^6^ CFUs) was resuspended in 10 mL MRS liquid medium, incubated in a shaking machine at 37℃ for 12 h, centrifuged at 4750 rpm for 10 min, about 100 µL bacterial supernatant was absorbed, and supplemented with methanol to 0.5 mL. After grinding by shock at low temperature, it was ultrasonic in ice bath for 10 min, and then stood at -20 ℃ for 30 min. After vortex mixing, it was centrifuged (12,000 rpm, 4 ℃, 5 min). After diluting the supernatant according to the actual situation, 100 µL of diluted liquid was taken into a 15 mL centrifuge tube. Pyridine, EDC solution and 3-NPH were added for derivatives. Used for LC-MS/MS detection and analysis. Meanwhile, the MRS liquid medium was used as a control for the LC-MS analysis.

Mouse feces: LC-MC analysis of SCFAs content in healthy mouse feces after gavage of Saline and *L.r*@(SA-CS)_2_ microgel respectively. During the experiment, the feces of the mice were collected after 7 d of gavage in each group, for a total of 5 feces in each group, and all items required for the experiment were autoclaved in advance. Weigh 50 mg of the sample and add an appropriate amount of 80% methanol-water solution. Introduce two steel balls for grinding, followed by centrifugation (20,000 rcf, 4 ℃) for 15 min. Transfer 20 µL of the supernatant into a 1.5 mL centrifuge tube and add an appropriate amount of EDC solution and 3-NPH for derivatization. Mix well with the initial mobile phase solution (500 µL), then transfer 200 µL into a sample vial for LC-MS/MS detection and analysis.

### Mouse short-chain fatty acid ELISA (SCFA) kit detection

The *L.r* or *L.r*@(SA-CS)_2_ (4.0 × 10^6^ CFUs) was resuspended in 10 mL MRS liquid medium, incubated in a shaking machine at 37 ℃ for 12 h, centrifuged at 4750 rpm for 10 min, about 100 µL bacterial supernatant was absorbed, and supplemented with methanol to 0.5 mL. After grinding by shock at low temperature, it was ultrasonic in ice bath for 10 min, and then stood at -20 ℃ for 30 min. After vortex mixing, it was centrifuged (12,000 rpm, 4 ℃, 5 min). After diluting the supernatant according to the actual situation, 100 µL of diluted liquid was taken into a 15 mL centrifuge tube. The Double-antibody one-step sandwich enzyme-linked immunosorbent assay (ELISA) was performed using micropores coated with SCFA antibodies. Specimen, standard, and HRP labeled detection antibodies were sequentially added to the pre-coated micropores, followed by warming and thorough washing. Color development was achieved by adding the substrate TMB, which underwent catalysis by peroxidase to produce a blue color that eventually turned yellow upon acidification. The intensity of the resulting color was directly proportional to the concentration of short-chain fatty acids (SCFA) in the sample. Absorbance (OD value) at a wavelength of 450 nm was measured using an enzyme-labeled instrument for calculation of sample concentration.

### 16 S rDNA sequencing and analysis

The 16 S rDNA sequencing and analysis were conducted at Lc-bio Technologies Co., Ltd (Hangzhou, China). A healthy mouse group served as the control, and three experimental groups were used: normal saline group, *L.r* group, and *L.r*@(SA-CS)_2_ group after modeling. During the experiment, the feces of the mice were collected after 7 d of gavage in each group, for a total of 3 feces in each group, and all items required for the experiment were autoclaved in advance. Sequencing of 16 S rDNA was performed on the collected samples.

### Tumor model establishment and therapeutic efficacy evaluation

Antitumor properties of *L.r*@(SA-CS)_2_ in CT26 tumor-bearing mice. Balb/c mice were anesthetized with isoflurane, a surgical incision was made in the left lower abdomen, the surgical incision was disinfected, and the cecum was removed from the surgical incision. An injection of 1.0 × 10^6^ colon cancer cells CT26-Fluc, suspended in 100 µL of normal saline, was given directly into the subserosa in the blood-rich region at the center of the cecum. Slow bolus injection should take less than 30 s. The needle was pulled out and pressed with a small cotton ball for one minute. After the injected fluid was absorbed by the intestinal wall, the cecum was reset to prevent pressurization and the abdominal cavity was closed. No antibiotics were used after the operation, which followed the principles of aseptic surgery. Routine feeding of the mice was performed after the modeling was completed.

CT26 orthotopic colon cancer was modeled 10 d after treatment began, and the time point of starting treatment was recorded as 0 d. Among the experimental groups were Saline, MK, *L.r*, *L.r*@(SA-CS)_2_, *L.r* + MK, and *L.r*@(SA-CS)_2_+MK. Saline, *L.r* and *L.r*@(SA-CS)_2_ were administered orally on 0 d, 3 d, 7 d, and 11 d, and MK on 4 d, 6 d, 8 d, 10 d, and 12 d. A dose of 4.0 × 10^8^ CFUs per one measurement were administered each time, while the dose of MK was 45 mg/kg and the control group received the same volume of Saline. During treatment, body weight and tumor size were measured every three days, and in vivo imaging was performed every five days. On 21 d, the mice were euthanized and the tumors and intestinal tissue were removed for subsequent testing.

### Clearance test for antibiotics

An orthopaedic colon cancer model has been successfully constructed in mice. Added compound antibiotic ABX (1 mg/mL ampicillin, 1 mg/mL neomycin trisulfate hydrate, 1 mg/mL metronidazole, 0.5 mg/mL vancomycin hydrochloride) to the drinking water of mice for a week to deplete the gut microbiota.

### The metabolic process of *L.r*@(SA-CS)_2_ microgel in vivo

To detect the presence of probiotics in the gastrointestinal tract, male Balb/c mice were gavaged with Saline, *L.r*, *L.r*@(SA-CS)_2_, and their feces were collected on 1 d, 3 d, 5 d, 7 d, 11 d, 15 d and 21 d. Following this, fecal samples were weighed and diluted with PBS at a consistent 10% dilution factor. Fecal dilutions were spread on solid agar plates and incubated for 24 h in a microbial incubator. Colonies from each sample were subsequently counted.

### Biosafety assessment of *L.r*@(SA-CS)_2_ microgels in vivo

For the in vivo evaluation of the microgel’s biosafety, ELISA kits were used to measure the changes in immune factors TNF-α, IFN-γ, and IL-6 levels in the blood of mice on 7 d and 20 d. On the 21 d, mice were euthanized and major organs were dissected for HE staining, including liver, kidney, spleen, lung, and heart. Furthermore, after intragastric administration of Saline, *L.r* and *L.r*@(SA-CS)_2_, blood was collected from the mice orbits at 0, 6 h, 12 h, 1 d, 7 d, and 30 d for routine WBC, RBC, PLT, blood biochemicals AST, ALT, and CERA further evaluated their biological safety.

### Statistical analysis

Analytical data were presented as mean ± standard deviation. One-way analysis of variance (ANOVA) was applied to the multi-group analysis, and Student’s t test was used to compare the two groups, with ns showing no significant difference. *p < 0.05, **p < 0.01, ***p < 0.001, ns means no statistical difference. Calculations were performed using GraphPad Prism 7. The results of fecal flora measured by 16 S rDNA sequencing were statistically analyzed by ASV.

## Results and discussion

### Preparation and characterization of *L.r*@(SA-CS)_2_ microgel

The microgel multilayer encapsulation system *L.r*@(SA-CS)_2_ was constructed by using the electrostatic interaction between SA and CS through layer-by-layer assembly and Ca^2+^ cross-linking. Prior to preparing the microgel, the optimal concentration of SA and CS for gel formation, as well as biosecurity, was first determined. Dose-dependent cytotoxicity and gelation with CS and SA concentrations. There was almost no cytotoxicity, when the concentration of SA and CS were less than 5 mg/mL (Figure S1A) and 8 mg/mL (Figure S1B), respectively. Since higher concentrations of CS had a certain bactericidal effect [28]. 5 mg/mL SA and 1 mg/mL CS were selected for subsequent studies based on comprehensive considerations.

**Fig. 1:**
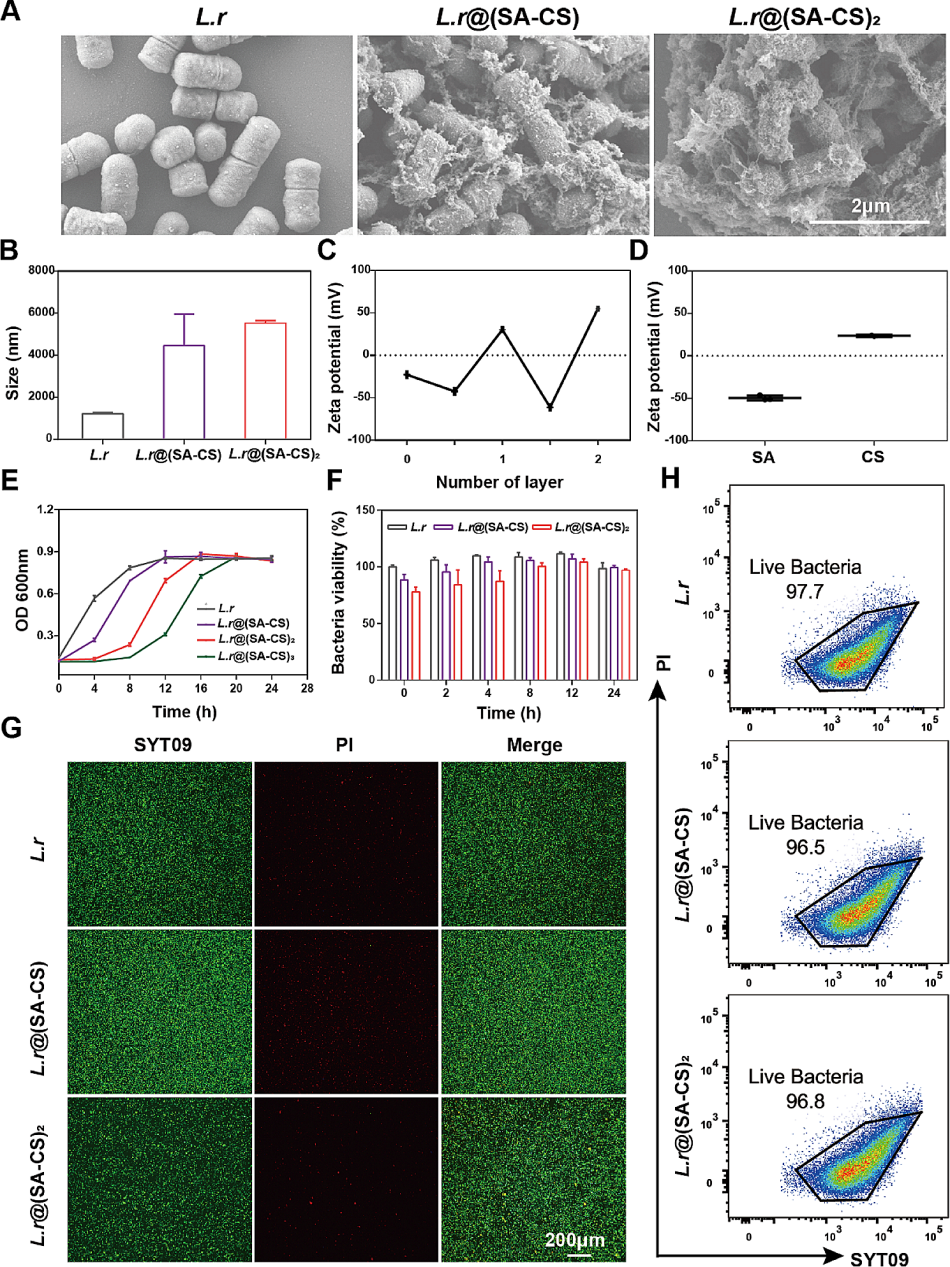
Characterization of *L.r*@(SA-CS)_2_. (**A**) Typical SEM images of *L.r*, *L.r*@(SA-CS) and *L.r*@(SA-CS)_2_. Scale bars, 2 μm. (**B**) Hydrodynamic diameter of *L.r*, *L.r*@(SA-CS) and *L.r*@(SA-CS)_2_ measured by dynamic light scattering. (**C**) Zeta potential of *L.r*, *L.r*@(SA-CS) and *L.r*@(SA-CS) _2_, respectively. (**D**) Zeta potential of SA and CS. (**E**) Growth curves of *L.r*, *L.r*@(SA-CS) and *L.r*@(SA-CS)_2_. Three types of *L.r* were cultured at 37 °C and OD 600 was measured at the indicated time points (*L.r*, 1 × 10^7^ CFUs/mL). (**F**) Viability analysis of *L.r*, *L.r*@(SA-CS) and *L.r*@(SA-CS)_2_ by AlamarBlue. Three types of *L.r* were cultured at 37 °C, and *L.r* viabilities were monitored by microplate reader at the indicated time (*L.r*, 1 × 10^7^ CFUs/mL). (**G**) Staining live-dead *L.r* of *L.r, L.r@*(SA-CS) and *L.r@*(SA-CS)_2_ analysed by Fluorescence microscope (*L.r*, 1 × 10^7^ CFUs/mL). Scale bars, 200 μm. (**H**) Staining live-dead *L.r* of *L.r, L.r*@(SA-CS) and *L.r*@(SA-CS)_2_ analysed by Flow cytometric (*n* = 3)

Scanning electron microscope (SEM) images showed that the unwrapped *L.r* was uniformly dispersed and had a smooth surface. After wrapping one layer of CS and SA, a thin film appeared on the surface of the *L.r*, which adhered to each other. Wrapping two layers, the *L.r* surface appeared thicker gel and more closely with each other (Fig. 1A). By dynamic light scattering (DLS), the particle diameter of *L.r*, *L.r@*(SA-CS), and *L.r*@(SA-CS)_2_ was 1217 nm, 4457 nm, and 5529 nm, respectively, was positively correlated increased with the number of coated layers (Fig. 1B). Moreover, the potential changes from − 22.7 mV to -42.4 mV after wrapping a layer of negatively charged SA around the surface of the unwrapped *L.r*. After wrapping the positively charged CS, the potential becomed + 30.3 mV. By repeating this process, the measured Zeta potential alternated between positive and negative values depending on the order in which SA or CS was added (Fig. 1C and D). Growth curves were recorded to determine whether the wrapping SA and CS had any effect on the activity of *L.r*. Despite our predictions, the more layers of wrapping have the opposite effect. As shown in Fig. 1E, after wrapping three layers of CS and SA, the logarithmic growth period of *L.r* was delayed by more than 12 h. Therefore, one and two layers wrapping were chosen for subsequent research to avoid the effect of multi-layer wrapping on the viability of *L.r* growth. Additionally, AlamarBlue was used to test the viability of *L.r*. Based on metabolic activity, AlamarBlue produced absorbance changes and fluorescence signals. Compared with *L.r*, *L.r@*(SA-CS) *and L.r@*(SA-CS)_2_ showed a slightly delayed activity before 8 h. Due to the slow release of *L.r* after wrapping, the activity value would continue to decrease as more *L.r* were released. *L.r*’s activity gradually increased among the three groups after 8 h, which indicated that as the number of *L.r* released increased, the activity of *L.r* reached the normal range of activity (Fig. 1F), so SA and CS encapsulation would not have a significant impact on *L.r*’s activity. The encapsulation effect was further verified by staining the live-dead *L.r* with microgels. As shown in Fig. 1G, there was no significant difference in green fluorescence between the unwrapped and encapsulated *L.r*, indicating that the microgel has almost no effect on the survival of *L.r*. Flow cytometry results demonstrated that the number of viable *L.r* in the *L.r, L.r@*(SA-CS) *and L.r@*(SA-CS)_2_ groups was 97.7%, 96.5%, and 96.8% (Fig. 1H), respectively, proving that microgel wrap was successful in converting *L.r* into cultures. Initially, the viability of the encapsulated *L.r* was weak, but as the number of released *L.r* increased, the viability gradually reached the same value as the bare *L.r*. At the same time, the microgel also maintained a high *L.r* viability, allowing for successful in vivo detection.

### Stability of *L.r*@(SA-CS)_2_ microgel in vitro and vivo gastrointestinal environment

**Fig. 2:**
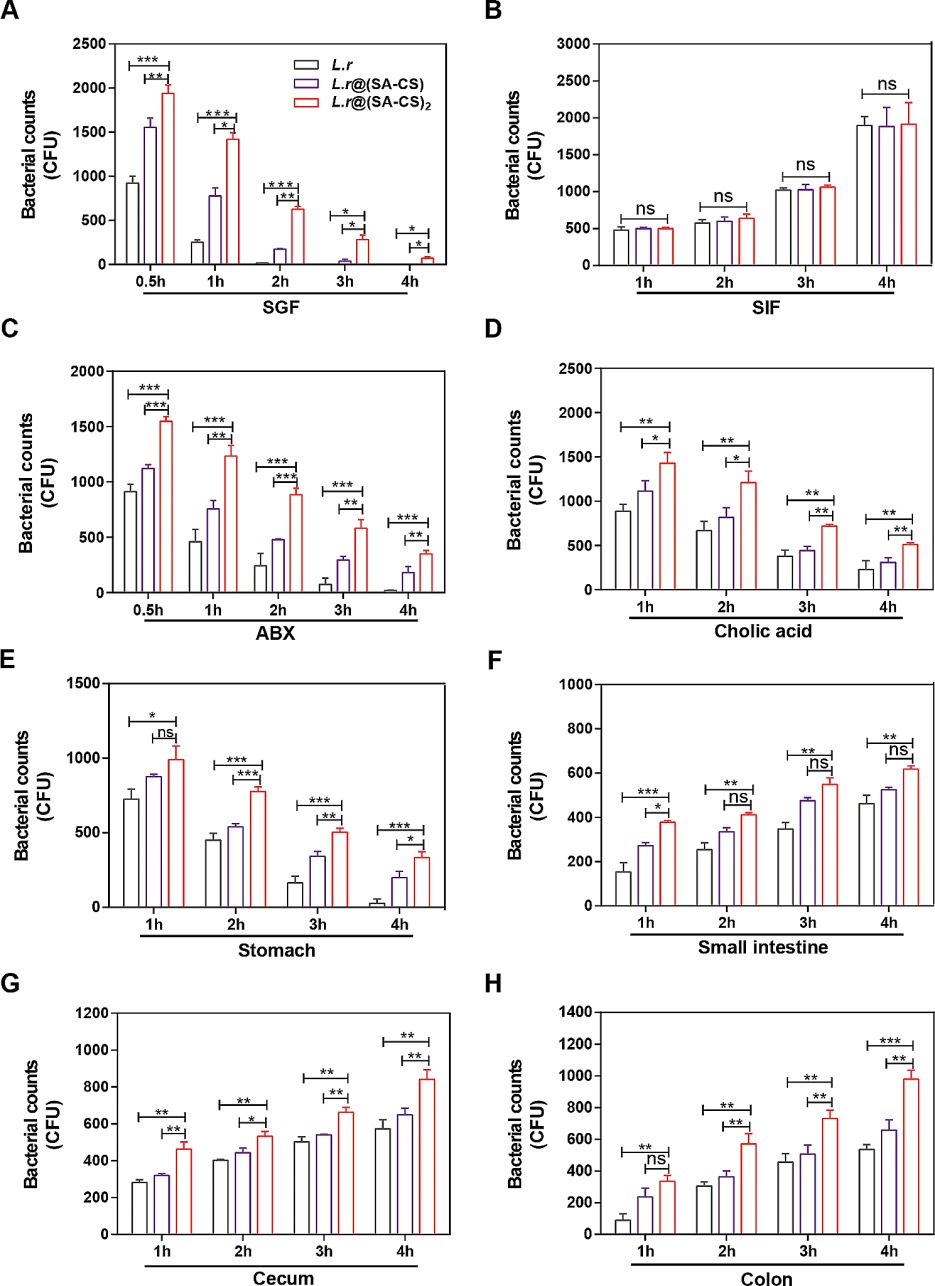
Stability of *L.r*@(SA-CS)_2_ microgel in vitro an *vivo* gastrointestinal environment. (**A** to **D**) Survivals of *L.r, L.r*@(SA-CS) and *L.r*@(SA-CS)_2_ (*L, r*, 4.0 × 10^5^ CFUs) after treatment with (A) SGF (1mL, pH 1.2, containing 2 g sodium chloride, 3.2 g pepsin, 7 mL hydrochloric acid per 1 L water), (**B**) SIF (1mL, pH 6.8, containing 0.2 M sodium hydroxide solution, 6.8 g potassium dihydrogen phosphate, 10 g trypsin per 1 L of water), (**C**) ABX (1mL, 1 mg/mL ampicillin, 1 mg/mL neomycin trisulfate hydrate, 1 mg/mL metronidazole, 0.5 mg/mL vancomycin hydrochloride), or (**D**) Cholic acid in vitro (1mL, 0.3 mg/mL) (*n* = 3). (**E** to **H**) Counts of *L.r* in the (**E**) stomach, (**F**) small intestine, (**G**) cecum, and (**H**) colon at the indicated time points after gavage of 4.0 × 10^8^ CFUs of *L.r, L.r*@(SA-CS) and *L.r*@(SA-CS)_2_ in vivo (*n* = 3). *p < 0.05, **p < 0.01, ***p < 0.001, ns means no statistical difference

The harsh environment of the gastrointestinal tract deactivates large amounts of *L.r*. after oral administration. Gastric juices, digestive enzymes, and bile salts may destroy microgels, penetrate pores, and degrade probiotics [33, 34]. For microgels, it is crucial to better utilize oral probiotics to keep *L.r* from being influenced by excessive gastric juices when entering the intestinal tract. Because of this, we tested the effect of gel coatings on the survival and growth of *L.r* in a gastrointestinal environment. In artificial gastric juice (SGF, pH 2.0), it appeared that the unwrapped *L.r* would immediately die, and that *L.r* had lost their vitality in two hours when the number of *L.r* in the *L.r*@ (SA-CS)_2_ group was 900 times greater than that of unwrapped group. The existence of *L.r* in one layer in gastric juice was only possible for 3 h, while *L.r* in two layers was possible for 4 h (Fig. 2A). In the artificial intestinal fluid (SIF, PH 6.8), the increasing trend of the number of *L.r* in three groups is similar. (Fig. 2B). After the administration of antibiotic solution, *L.r*@(SA-CS)_2_ group still had a higher *L.r* survival rate than others. Four hours later, the number of *L.r* in the two layers group was 2 times higher than the single layer group and 20 times higher than the unwrapped group (Fig. 2C). Counts of *L.r* in Cholic acid gradually decreased over time in group *L.r*, *L.r*@(SA-CS) and *L.r*@(SA-CS)_2_, with reduction rates of 86%, 80%, and 70% respectively (Fig. 2D). As shown in Fig. 2E, using diluted and counted gastric juice samples, the amount of *L.r* wrapped in two layers was 2 times greater than in one layer and 12 times greater than in the unwrapped layer, indicating that the microcapsules provide superior protection against gastric juices. At the same time, three groups of *L.r* could all increase with the extension of residence time in the small intestinal fluid, cecal fluid and colonic fluid, (Fig. 2F-H). Figure S2 was representative photographs of solid MRS agar plates of all *L.r* diluted and coated in vitro and in vivo experiments. Based on the results of the internal and external gastrointestinal environment experiments, there was high resistance to gastric juice, ABX, and Cholic acid in *L.r*@(SA-CS)_2_ microcapsules, and the amount of unwrapped *L.r* in intestinal juice also increased with time, indicating that *L.r* itself could withstand intestinal juice and survive.

On the side, we observed the morphological changes of *L.r* and *L.r*@(SA-CS)_2_ in SGF, SIF, and ABX for 2 h using scanning electron microscopy. In SGF, the majority of the bare *L.r* fractured and perished, while in ABX, the bacterial wall contracted and deformed. However, in SIF, the bacterial morphology remained largely unchanged. Upon microgel encapsulation, the *L.r* retained their morphological integrity in SGF, SIF, and ABX, and the gel encapsulation was evident. This demonstrated that our encapsulation system provided a protective function. Furthermore, compared to SGF and ABX, the gel state in SIF exhibited signs of swelling and cracking, further indicating that our material facilitates pH-responsive bacterial release (Figure S3). Therefore *L.r*@(SA-CS)_2_ with stronger resistance was selected for follow-up experiments, was able to reduce *L.r* death from gastric passage and improve *L.r* stability and viability in various gastrointestinal fluid environments.

### SCFAs Produced by *L.r*@(SA-CS)_2_ microgel elicited apoptosis of CT26 Cells

**Fig. 3:**
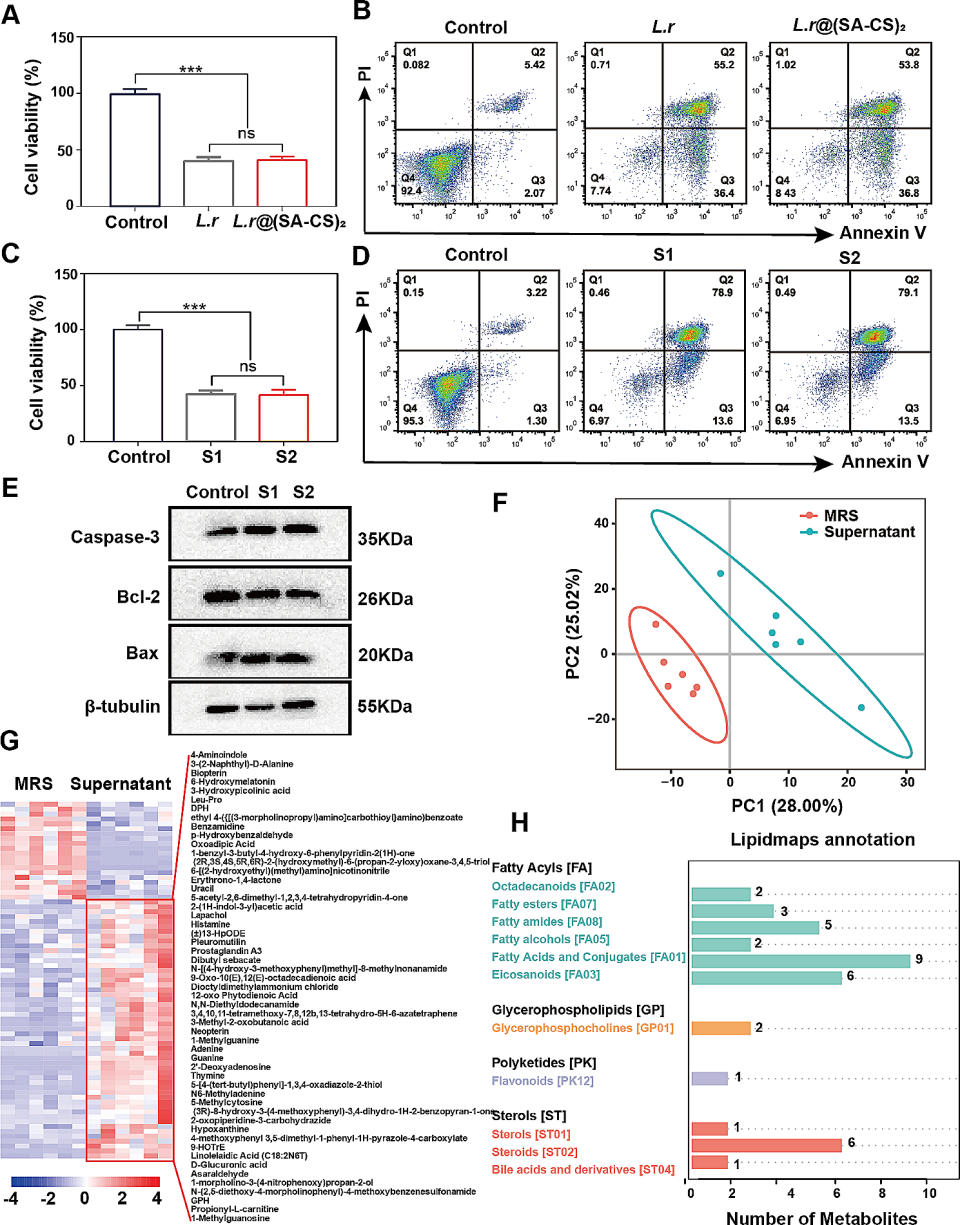
SCFAs produced by *L.r*@(SA-CS)_2_ microgel elicited apoptosis of CT26 cells. (**A**) The viability of *L.r*, *L.r*@(SA-CS)_2_ and CT26 cells was determined by CCK-8 after a 10 h incubation. (**B**) A flow cytometry method using Annexin V-FITC/PI for detecting cell apoptosis in co-cultured *L.r* and cells (*n* = 3). (**C**) CCK8 detected the survival rate of the supernatant of *L.r*, *L.r*@(SA-CS)_2_ and CT26 cells after co-incubation for 10 h. S1: Supernatant collected after *L.r* was shaken at 37 °C for 12 h, S2: Supernatant collected after *L.r*@(SA-CS)_2_ was shaken at 37 °C for 12 h (*n* = 3). (**D**) Annexin V-FITC/PI flow cytometry was used to detect the apoptosis of cells after co-incubation of supernatant and cells (*n* = 3). (**E**) Expression of apoptosis-related proteins: Caspase-3, Bax, Bcl-2 after co-incubation with cells in different groups. (**F**) PLS-DA analysis of MRS liquid medium and *L.r* supernatant by LC-MS (*n* = 6). (**G**) Heat map of differential metabolites. The abscissa is the sample, and the ordinate is the metabolite of the differential expression. Different colors indicate different expression levels of metabolites. The color range from blue to yellow to red represents the value of the abundance from low to high. The red color indicates highly expressed metabolites. Colors indicate metabolites with low expression. (**H**) Lipidmaps annotation classification map of differential metabolites, the abscissa represents the number of metabolites, and the ordinate represents the eight categories of lipids annotated in Lipidmaps. *p < 0.05, ***p < 0.001, ns means no statistical difference

Various mechanisms have been identified for the action of *L.r*, a widely studied probiotic [15]. Hence, in order to determine whether *L.r* had an antitumor effect, *L.r* and *L.r*@(SA-CS)_2_ were cultured with CT26 cells. As compared with the control group, both *L.r* and *L.r*@(SA-CS)_2_ in the experimental group were able to induce tumor cell death and the level of cell death did not differ significantly between the groups (Fig. 3A). The Annexin V-FITC/PI flow staining was used to quantitatively evaluate tumor apoptosis caused by *L.r* and *L.r*@(SA-CS)_2_. As shown in Fig. 3B, the total apoptosis rates of CT-26 cells induced by *L.r* and *L.r*@(SA-CS)_2_ bacteria were 91.6% and 90.6%, *L.r* is able to induce apoptosis in tumor cells and microgel does not significantly interfere with *L.r* performance. Despite the fact that *L.r*. can induce apoptosis in tumor cells, the precise mechanism of this process is still unknown. Therefore, the next step was to determine which component of *L.r* causes tumor cell death, and whether this component is produced by bacterial metabolism. Supernatants of *L.r* were tested to test this conjecture. As shown in Fig. 3C, the supernatants of both groups *L.r* and *L.r*@(SA-CS)_2_ induced apoptosis of tumor cells. After flow cytometry was used to verify apoptosis induction, the results showed that both groups had nearly similar results, 92.5% and 92.6%, respectively (Fig. 3D). Meanwhile, the results of Western blotting analysis showed that the supernatant of *L.r* culture and cells would change relevant apoptotic proteins (Fig. 3E), and quantitative analysis indicated that the pro-apoptotic proteins Caspase-3 and Bax increased, and the anti-apoptotic protein Bcl-2 decreased (Figure S4A). Additionally, the Bcl-2/Bax ratio was employed as an indicator of cellular anti-apoptotic capability (Figure S4B). It is worth mentioning that the activated form of the apoptotic protein caspase-3, Cleaved-caspase 3, induced by the supernatant was also increased (Figure S4C, D). Based on the above results, *L.r* may increase the pro-apoptotic protein Caspase-3, Bax and decrease the anti-apoptotic protein Bcl-2 through the production of certain metabolites, which may lead to the apoptosis of tumor cells.

To further explore the therapeutic mechanism of *L.r*, metabolomics analysis was performed on the supernatant of *L.r*. As can be seen in Fig. 3F, the samples in the MRS and Supernatant groups were relatively concentrated, suggesting that the differences between the samples were large compared to the differences within them. Significant differences were observed between the Supernatant and MRS groups. A heatmap can be seen in Fig. 3G to visualize the differences in the abundances of those metabolites in the different treatments based on their similar expression profiles in the sample. The components of the base, highly expressed metabolites in the supernatant after *L.r* culture. The metabolites identified in each group were then annotated both functionally and taxonomically. The Lipidmaps annotation and classification results for the metabolites were shown in Fig. 3H, through the metabolomic analysis of the culture supernatant of *L.r*, compared to MRS Medium, fatty acids and their conjugates exhibited the most significant disparity in metabolite content, with the utmost divergence observed in short-chain fatty acids (SCFAs). Previous studies have demonstrated that both acetic acidand propionic acid possess potential antitumor properties [1, 35]. Thus, the results of the experiments showed that *L.r*@(SA-CS)_2_ microgels induce apoptosis of tumor cells through the production of SCFAs. It is worth mentioning that, as shown in Figure S5, the production of SCFAs by *L.r* is initially limited within 8 h due to the gel system coating. However, as more *L.r* are released, the yield of SCFAs increases and eventually reaches a level comparable to that of bare *L.r*. Therefore, microgel encapsulation does not significantly affect the ability of *L.r* to release SCFAs.

### *L.r*@(SA-CS)_2_ microgel relied on intestinal flora to play an antitumor role in Vivo

**Fig. 4:**
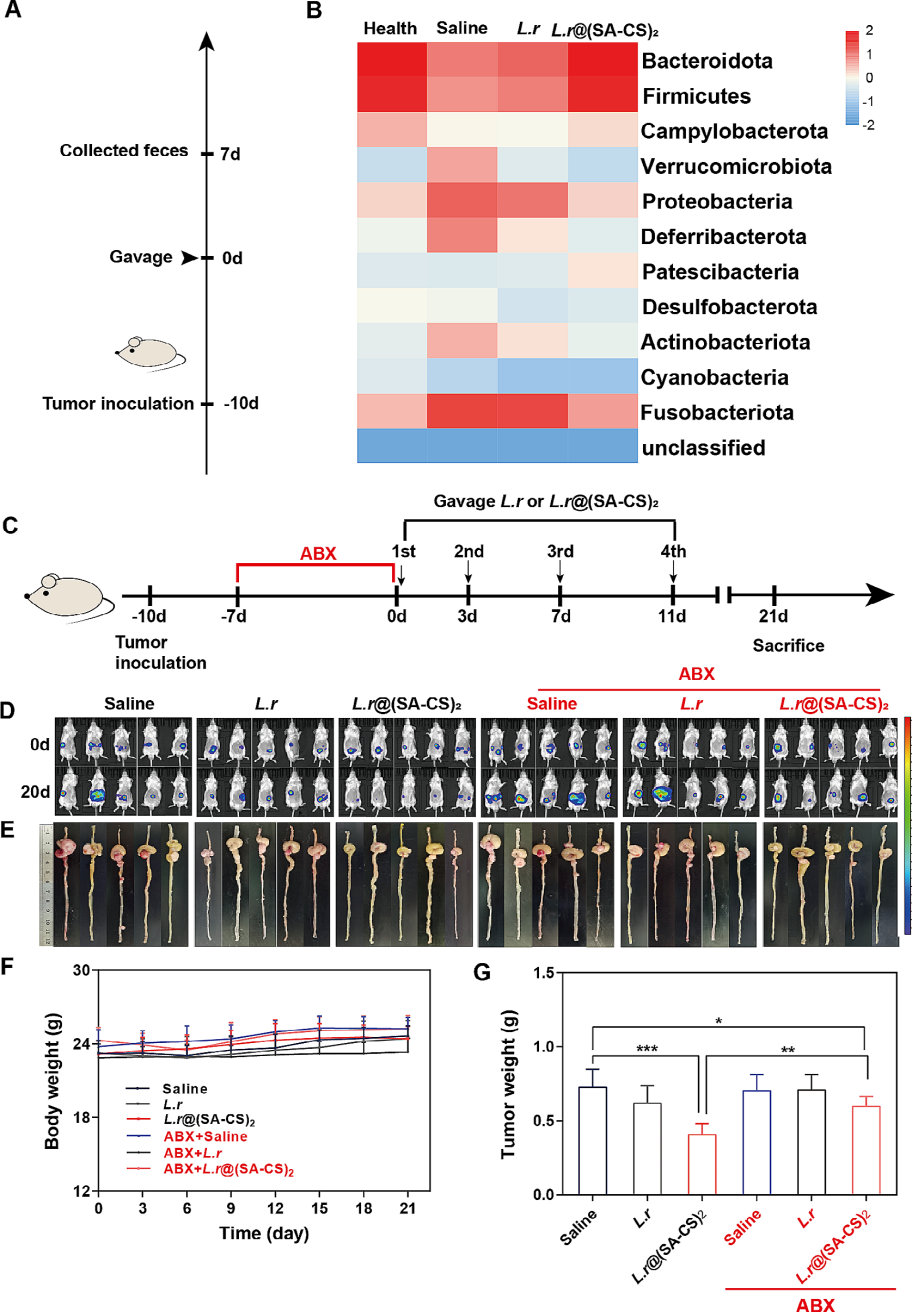
*L.r*@(SA-CS)_2_ microgel relied on intestinal flora to play an antitumor role in vivo. (**A**) Scheme of 16 S rDNA Sequencing detection feces flora changes in Health mice, and Saline, *L.r*, *L.r*@(SA-CS)_2_ in CT26 tumor-bearing mice on 7 d after gavage (100µL, *L.r*, 4.0 × 10^8^ CFUs). (**B**) Heatmap analysis, each row represents a species and each column represents a sample or grouping. The plot uses a blue-to-red gradient to reflect the change in abundance from low to high. (*n* = 3). (**C**) A schematic diagram illustrating the clearance of antibiotics. (**D**) An in vivo imaging study of all mice aged 0 d and 21 d in each group were performed. (**E**) Images of all colonic tumors after euthanasia in 21 d mice. (**F**) Weight curves of all mice in each group after 21 d. (**G**) Weight records of all mice in each group with tumors (*n* = 6). *p < 0.05, **p < 0.01, ***p < 0.001

Having explored the mechanism of microgel-induced apoptosis of tumor cells in the previous section, we proceed to explore how oral microgels affect gut flora in the hindgut. After probiotics are administered orally, the gut flora is the first to be altered [5]. Therefore, after gavage of microgel to mice, the 16 S rDNA test was performed first on their feces in order to determine the changes in flora (Fig. 4A). The colon cancer mice group showed significant increases in harmful bacteria such as *Proteobacteria and Fusobacteriota*, in comparison to the healthy mice group. *L.r* group intragastric administration was similar to saline group intragastric administration in terms of flora composition (Fig. 4B). Although the proportion of intestinal flora after oral administration of *L.r*@(SA-CS)_2_ microgel was not entirely comparable to that of healthy mice, it was very close to the composition of intestinal flora of healthy mice compared with the group treated only with intragastric administration of saline, indicating that oral administration of *L.r*@(SA-CS)_2_ microgel played a significant role in regulating intestinal flora. Moreover, the intestinal flora of the mice changed significantly after intragastric administration of microgel, mainly due to reduced harmful bacteria such as *Proteobacteria* and *Fusobacteriota*, and increased beneficial bacteria such as *Bacteroidota* and *Firmicutes*. It might be considered that the production of SCFAs lowers the pH value of the intestinal tract, making the intestinal tract more resistant to colonization by harmful bacteria, while encouraging probiotic production and growth [36–38]. Based on the results, butyric acid-producing beneficial bacteria were mainly induced by microgel. Such as, the enrichment of *Muribaculaceae* can enhance the abundance of intestinal microbiota, thereby increasing butyrate production in plasma and feces [39]. In constipated rats, rhubarb can elevate the levels of intestinal probiotic *Ligilactobacillus*, facilitate fiber degradation and SCFAs production, thus playing a crucial role in maintaining intestinal stability [40]. A study has demonstrated that ginseng polysaccharides have the ability to modulate gut flora composition by promoting an increase in *Muribaculum* abundance, consequently leading to enhanced SCFAs production [41]. Furthermore, an augmented presence of *Muribaculum* within the intestinal flora promotes elevated butyric acid production which is involved in repairing the integrity of the intestinal epithelial barrier and reducing inflammatory responses [42].

In order to validate the efficacy of oral *L.r*@(SA-CS)_2_ microgel in enhancing butyric acid levels in mice, we gavaged Saline and *L.r*@(SA-CS)_2_ microgel to healthy mice. It can be seen that the SCFAs in the mice were significantly increased after gavage of *L.r*@(SA-CS)_2_ microgel (Figure S6A). Among them, acetic acid increased the most, followed by butyric acid, propionic acid, valeric acid, isobutyric acid, isovaleric acid and hexanoic acid (Figure S6B-H). The abundance of acetic acid as the most prevalent organic acid has been demonstrated by numerous studies, indicating its production by various bacteria to serve as an energy source for gut flora. Butyric acid, however, is generally produced by certain bacteria. It is worth our attention, after taking the microgel orally, the mice more than tripled their butyric acid production, other SCFAs did not seem to change significantly. Therefore, this experiment once again confirmed the ability of oral *L.r*@(SA-CS)_2_ microgel to effectively regulate the intestinal flora, leading to an increase in the population of butyric acid-producing bacteria in the gut and subsequently enhancing butyric acid production.

In addition, we also observed in the experiment that the tumor growth of mice in the microgel group was slow, which might be related to the intestinal flora. To test this hypothesis, we set up an in-situ colon cancer model in mice, which were pre-treated with antibiotics for a week to eliminate the flora in the body, to see if the anti-tumor effect of *L.r*. The schematic diagram of Fig. 4C showed that after weeks of antibiotics to deplete the intestinal flora, *L.r* and microgel were administered orally on 0 d, 3 d, 7 d, 11 d, and mice were imaged in vivo on 0 d, 20 d (Fig. 4D), followed by photographic documentation of tumor tissues on 21 d after euthanasia (Fig. 4E). The body weight of the mice in each group did not change significantly throughout the experiment (Fig. 4F). The results suggested that tumors in mice pretreated with antibiotics grew overall faster than tumors in mice not pretreated with antibiotics. In contrast to the saline group, the inhibition rate of the *L.r*@(SA-CS)_2_ group was 44%, whereas the inhibition rate of the *L.r* group was 15%, indicating the microgel did inhibit tumor growth. Following pretreatment with antibiotics, the tumor inhibition rate for the *L.r*@(SA-CS)_2_ group became 18%, proving that gut flora influenced microgel antitumor effects in vivo. *L.r* could directly produce SCFAs mentionted, which might explain why tumors in ABX + *L.r*@(SA-CS)_2_ were still smaller than those in *L.r*. This further indicated that the microgel entered the intestinal tract and metabolized with SCFAs, as some tumors were reduced in the ABX + *L.r*@(SA-CS)_2_ group in comparison to the saline group (Fig. 4G). As shown in Figure S7A, by observing the metabolism of *L.r* in the body after intragastric administration of saline to the mice, *L.r* and *L.r*@(SA-CS)_2_, the number of *L.r* spread in the feces of the two groups gradually decreased with time, compared to the saline group. The *L.r* group administered by intragastric administration had a 1.5 times higher rate of *L.r* metabolism than the microgel group, further highlighting the benefits of microgel. Besides protecting *L.r* from gastric acid erosion, microgels could slow down their metabolic processes, stay in the intestinal tract for a long time, maintain high probiotic flora, and reduce the times of feeding *L.r*. As the SA and CS degrades, more *L.r* were released in the intestinal tract, producing a better anti-tumor effect and providing a basis for subsequent drug efficacy testing. Representative photographs of solid MRS agar plates of *L.r* were shown in Figure S7B.

### Augmented antitumor efficacy by *L.r*@(SA-CS)_2_ microgel in combination with MK

Although microgels regulate the gut flora and produce SCFAs, their anti-tumor effects are still limited. Previous experiments have shown that SCFAs produced by microgels increased gut butyrate-producing bacteria through intertrophy. Among the most important SCFAs, butyric acid is synthesized by intestinal microorganisms as they ferment dietary fiber, protein, and polypeptide [11]. As a nutrient factor in the colon, butyric acid has an important impact on colonic mucosa proliferation and inhibits DNA synthesis in the G1 phase of the cell cycle [17]. In colon cancer, the low expression of butyrate receptor GPR109A on the surface of the colon epithelium prevents the apoptosis of the alienated colon cells and accelerates tumor growth [18]. It is intended to investigate whether drug agonists that activate the expression of the GPR109A receptor can enhance the antitumor effects of microgels. To test this hypothesis, we combined the microgel with MK, an agonist of the GPR109A receptor. As butyric acid is unstable and easily volatile, sodium butyrate, a stable compound of butyric acid, is often used as an in vitro model to verify its function [11]. Therefore, the results of the CCK-8 experiment indicated that sodium butyrate killed CT26 concentration-dependently (Figure S8A). As shown in Figure S8B, the pro-apoptotic proteins Caspase-3 and Bax increased and the anti-apoptotic protein Bcl-2 decreased after apoptosis occurred. It is worth mentioning that, due to the activation of the apoptotic protein caspase-3, the expression of its activated form, Cleaved-caspase 3, was also increased. Based on the results of the CCK-8 study, it was found that MK alone had a negligible level of cytotoxicity (Figure S8C), but the combination of MK with sodium butyrate showed a significant cytostatic effect (Figure S8D). As a result, in vitro experiments demonstrated that sodium butyrate in combination with MK did indeed have anti-tumor effects.

**Fig. 5:**
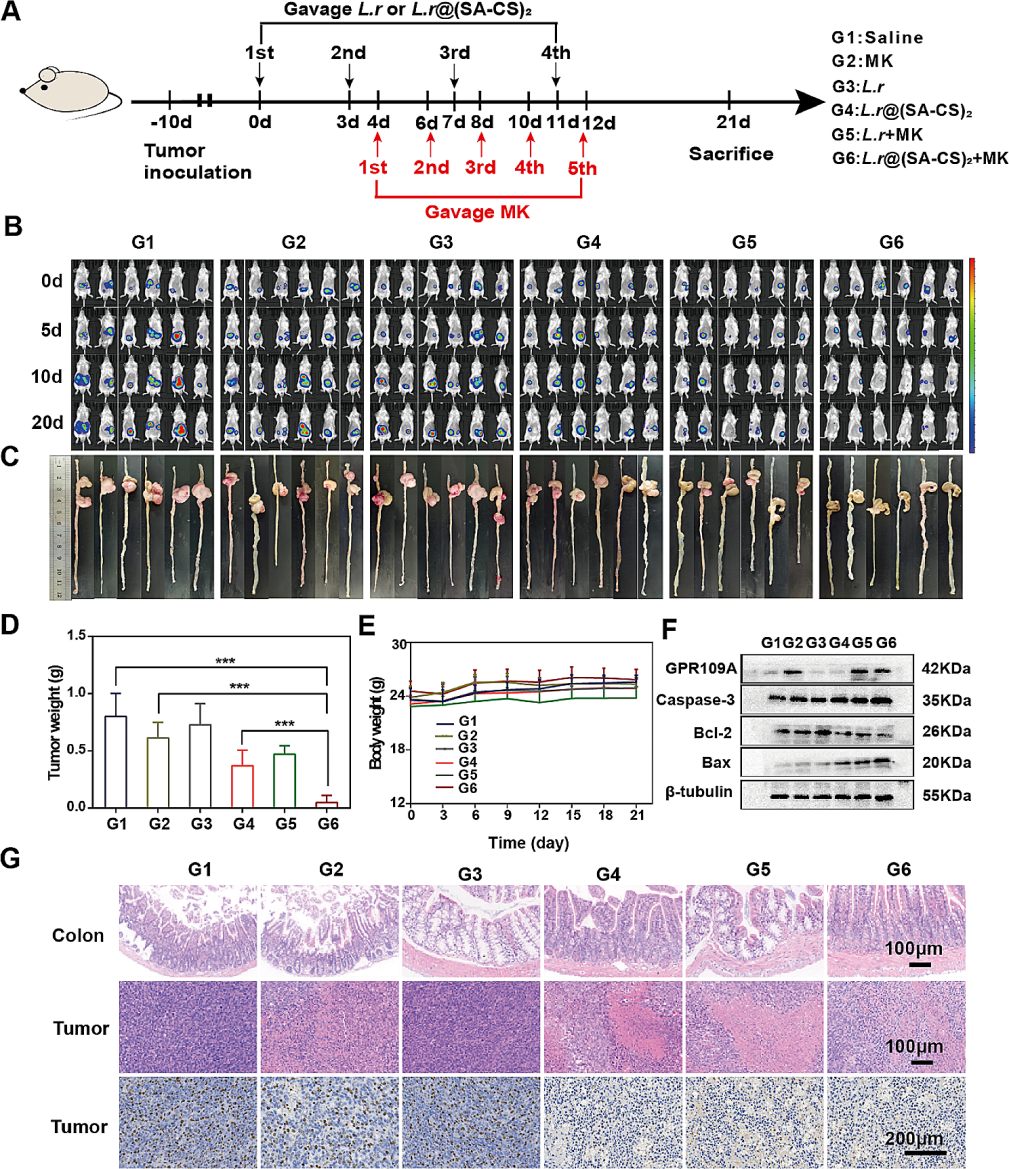
Augmented antitumor efficacy by *L.r*@(SA-CS)_2_ microgel in combination with MK. (**A**) Schematic of experimental design. (**B**) To observe tumor growth in 0 d, 5 d, 10 d, and 20 d mice, live imaging is performed (D-luciferin, 15 mg/mL, 150 mg/kg). G1: Saline, G2: MK, G3: *L.r*, G4: *L.r*@(SA-CS)_2_, G5: *L.r* + MK, G6: *L.r*@(SA-CS)_2_ +MK (100µL, *L.r*, 4.0 × 10^8^ CFUs; MK:45 mg/kg). (**C**) Tumor photographs taken after euthanasia in 21 d mice. (**D**) Records of tumor weights in each group of mice. (**E**) Records of body weight curves for each group of mice. (**F**) The expression of apoptosis proteins in tumor tissues of each group was detected by WB (*n* = 3). (**G**) After 21 d of treatment, HE staining of colon and tumor tissue and Ki67 staining of tumor tissue were performed on each group of mice (*n* = 3). ***p < 0.001

In-vivo anti-tumor experimental process of microgels in combination with MK was shown in Fig. 5A. Tumor growth was monitored by mice imaging (Fig. 5B). On 21 d, the cecum and the following intestinal tissue of mice were removed and photographed as shown in Fig. 5C, tumor tissues were stripped from the surface of the intestine, and tumor weight was recorded (Fig. 5D). *L.r*@(SA-CS)_2_+MK group had the best therapeutic effect. Compared with the control group, the tumor inhibition rate of *L.r*@(SA-CS)_2_+MK group was 93%, which showed obvious tumor inhibition effect. Compared with *L.r*@(SA-CS)_2_ group, the tumors in *L.r*@(SA-CS)_2_+MK group were smaller and statistically significant (*p* < 0.001). The tumor inhibition rate of *L.r* + MK was 40%, which was significantly lower than that of *L.r*@(SA-CS)_2_+MK group. During the treatment, the body weights of mice in each group were recorded (Fig. 5E), and no significant weight loss was observed in all groups, indicating that the microgel had good biological safety. The WB results of tumor tissue were shown in Fig. 5F. In *L.r*@(SA-CS)_2_, *L.r* + MK and *L.r*@(SA-CS)_2_+MK groups, pro-apoptotic protein Caspase-3 and Bax were increased, while anti-apoptotic protein Bcl-2 was decreased. MK, *L.r* + MK, and *L.r*@(SA-CS)_2_+MK groups taking MK had higher expression of GPR109A receptors. The levels of Cleaved-caspase 3 protein in tumor tissues of each group can also improve the fact (Figure S9A, B). The expression of pro-apoptotic protein was the highest in *L.r*@(SA-CS)_2_ microgel combined with MK group, while the anti-apoptotic protein was the lowest. Colon and tumor tissue were stained with HE and Ki67 following euthanization of 21 d mice. As could be seen from Fig. 5G, the colonic mucosa of the control group had significant damage, whereas the mucosa of the *L.r*@(SA-CS)_2_+MK was relatively more complete and better restored. There was no significant cell death in the tumor tissue of the control group, but nuclear pyknosis was evident in the tumor tissue of the treated group, and the cells were loosely arranged, indicating substantial cell death, with Ki67 showing the least number of proliferating cells.

### Biosafety assessment of *L.r*@(SA-CS)_2_ microgel

**Fig. 6:**
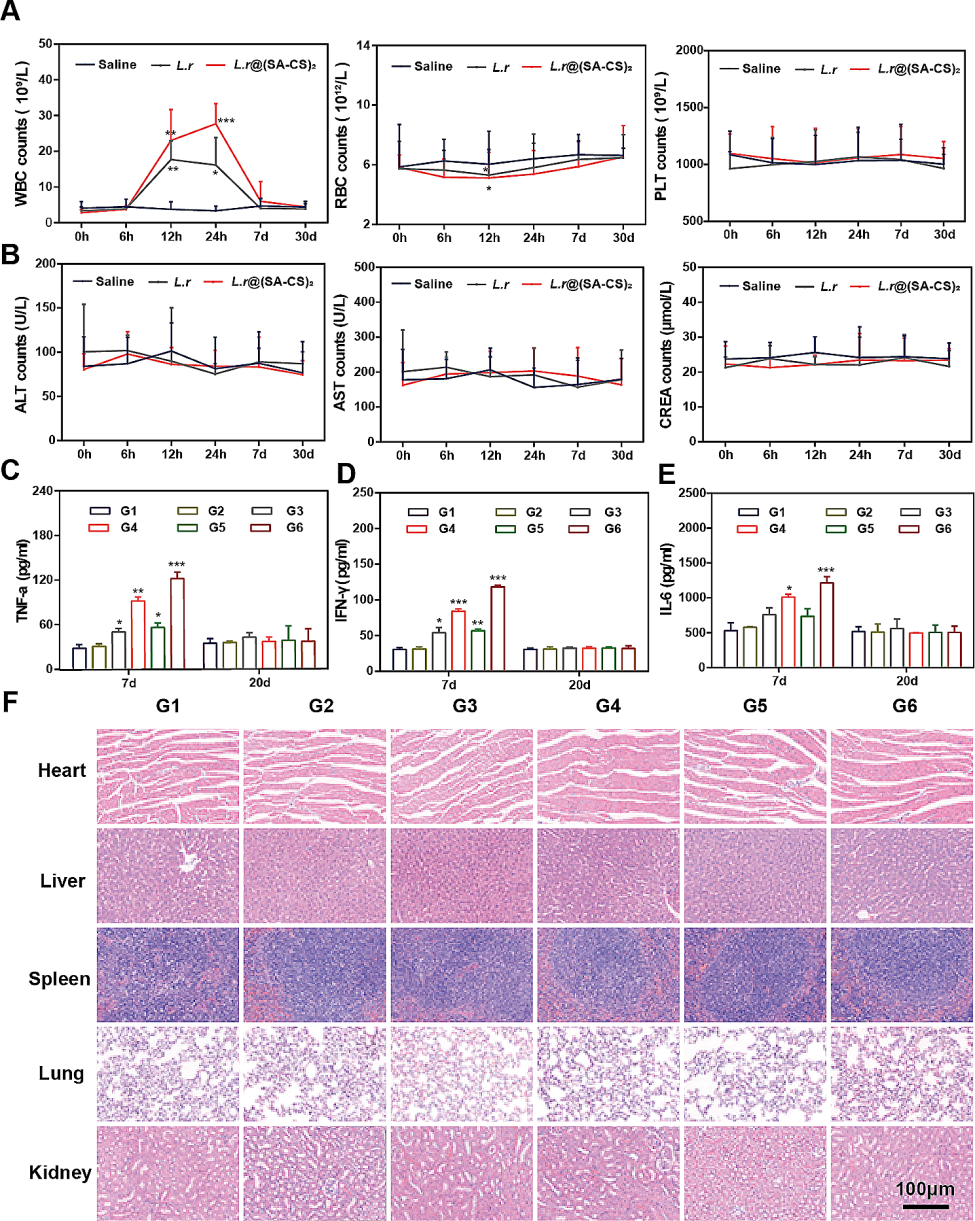
Biosafety assessment of *L.r*@(SA-CS)_2_ microgel. Saline, *L.r*, *L.r*@(SA-CS)_2_ (100 µL, 4.0 × 10^8^ CFUs) were intragastrically administered, and orbital blood was collected at designated time points (0, 6 h, 12 h, 1 d, 7 d, 30 d). (**A**) Blood routine analysis, including WBC, RBC, and PLT; (**B**) Blood biochemistry: AST, ALT, and CERA levels (*n* = 6). Reference range of WBC counts: 0.8–6.8 10^9^/L RBC counts: 6.36–9.42 10^12^/L; PLT counts: 450–1590 10^9^/L; ALT counts: 10.06–96.47 U/L; AST counts: 36.31-235.48 U/L; and CREA counts 10.91–85.09 µmol/L. (**C**) TNF-α, (**D**) IFN-γ, (**E**) IL-6 level in blood serum after treatments. G1: Saline, G2: MK, G3: *L.r*, G4: *L.r*@(SA-CS)_2_, G5: *L.r* + MK, G6: *L.r*@(SA-CS)_2_ +MK. (**F**) Representative images of HE staining for major organs after treatments (*n* = 3). *p < 0.05, **p < 0.01, ***p < 0.001

The results of routine blood test were shown in Fig. 6A. WBC levels increased within 7 d after intragastric administration of *L.r* and *L.r*@(SA-CS)_2_, but gradually return to normal. Initially, the red blood cell count dropped, then returned to normal. It could be seen from the blood biochemical results in Fig. 6B, there was no significant difference between *L.r* and *L.r*@(SA-CS)_2_ in terms of PLT, ALT, AST, CERA etc. Most of the blood routine and blood biochemical data of mice treated with *L.r* and *L.r*@(SA-CS)_2_ were within normal limits compared with untreated healthy mice. Aside from the in vivo pharmacodynamics test, ELISA kits were used to detect changes in inflammatory factors levels on 7 d and 20 d. The results indicated TNF-α (Fig. 6C), IFN-γ (Fig. 6D), and IL-6 (Fig. 6E) increased in the group of intragastric bacteria on 7 d, but the indexes could return to normal levels at 20 d, showing oral bacteria-induced inflammation was tolerable without chronic toxicity. Throughout the important organs, including the heart, liver, spleen, lung and kidney, no apparent pathological changes in cellular tissue structure were found (Fig. 6F). This suggested good biocompatibility of the microgel.

## Conclusion

Here, the *L.r*@(SA-CS)_2_ microgel delivery system was successfully constructed by layer-by-layer assembly using the electrostatic interaction between SA and CS. Microgel-coated *L.r* has shown good gastrointestinal tolerance and significantly improved its oral availability. With the production of SCFAs, *L.r* promotes apoptosis of tumor cells while remodeling the gut flora, resulting in a reduction in harmful bacteria and an increase in beneficial bacteria that produce butyric acid. This is the first application of the butyric acid receptor agonist MK-6892 in a tumor model, where it significantly enhanced the anti-tumor effect of *L.r* and demonstrated a highly efficient inhibitory effect on in situ colon cancer. The biocompatible *L.r*@(SA-CS)_2_ microcapsules provide a new idea for oral probiotics in the treatment of solid tumors, and make the application of MK in tumor therapy possible, bringing a new treatment option for colon cancer. We hope to further explore the relationship between gut flora and short-chain fatty acids and propose new approaches to CRC prevention and treatment.

### Electronic supplementary material

Below is the link to the electronic supplementary material.

Supplementary Material 1

## Data Availability

The data that support the findings of this study are available from the corresponding author upon reasonable request.
