# Supplementary material for: Taking SCFAs produced by Lactobacillus reuteri orally reshapes gut microbiota and elicits antitumor responses
Source: J Nanobiotechnology. 2024 May 12;22:241. doi: 10.1186/s12951-024-02506-4 (PMC11089779; doi:10.1186/s12951-024-02506-4)
Supplement: Supplementary file 1 — Supplementary Material 1 [file 12951_2024_2506_MOESM1_ESM.docx]

Supporting Information of

**Taking SCFAs produced by *Lactobacillus reuteri* orally reshapes gut microbiota and elicits antitumor responses**

*Nannan Li^1,2#^, Lili Niu^1,2#^, Yao Liu^2,3#^, Yang Wang^1,2^, Xiaomin Su^1,2^, Ce Xu^1,2^, Zanya Sun^1,2^, Huishu Guo^1*^, Jingru Gong^2*^, and Shun Shen^2*^*
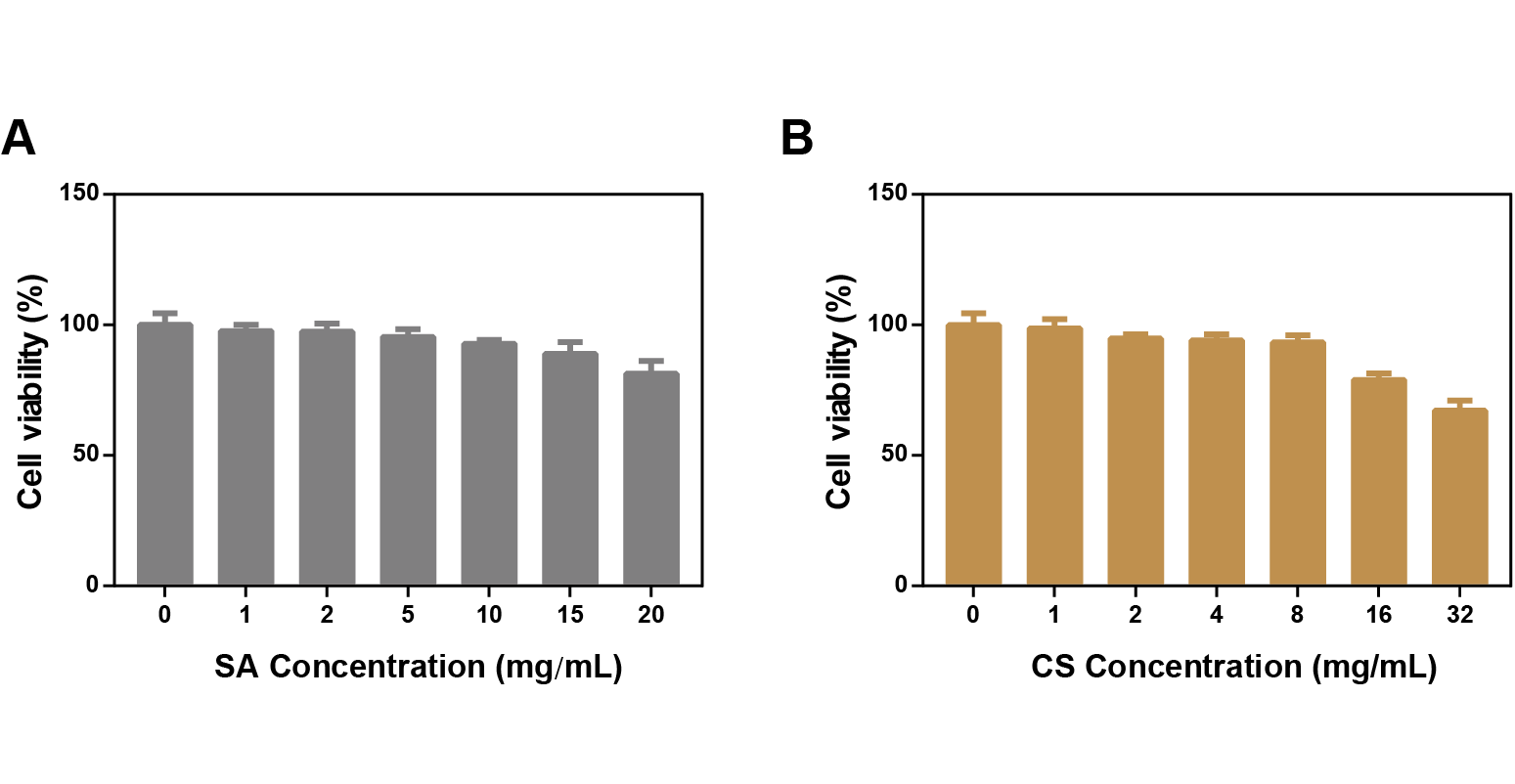


**Figure S1.** CCK-8 detected the effect of substances SA and CS on CT26 cells (n=5).


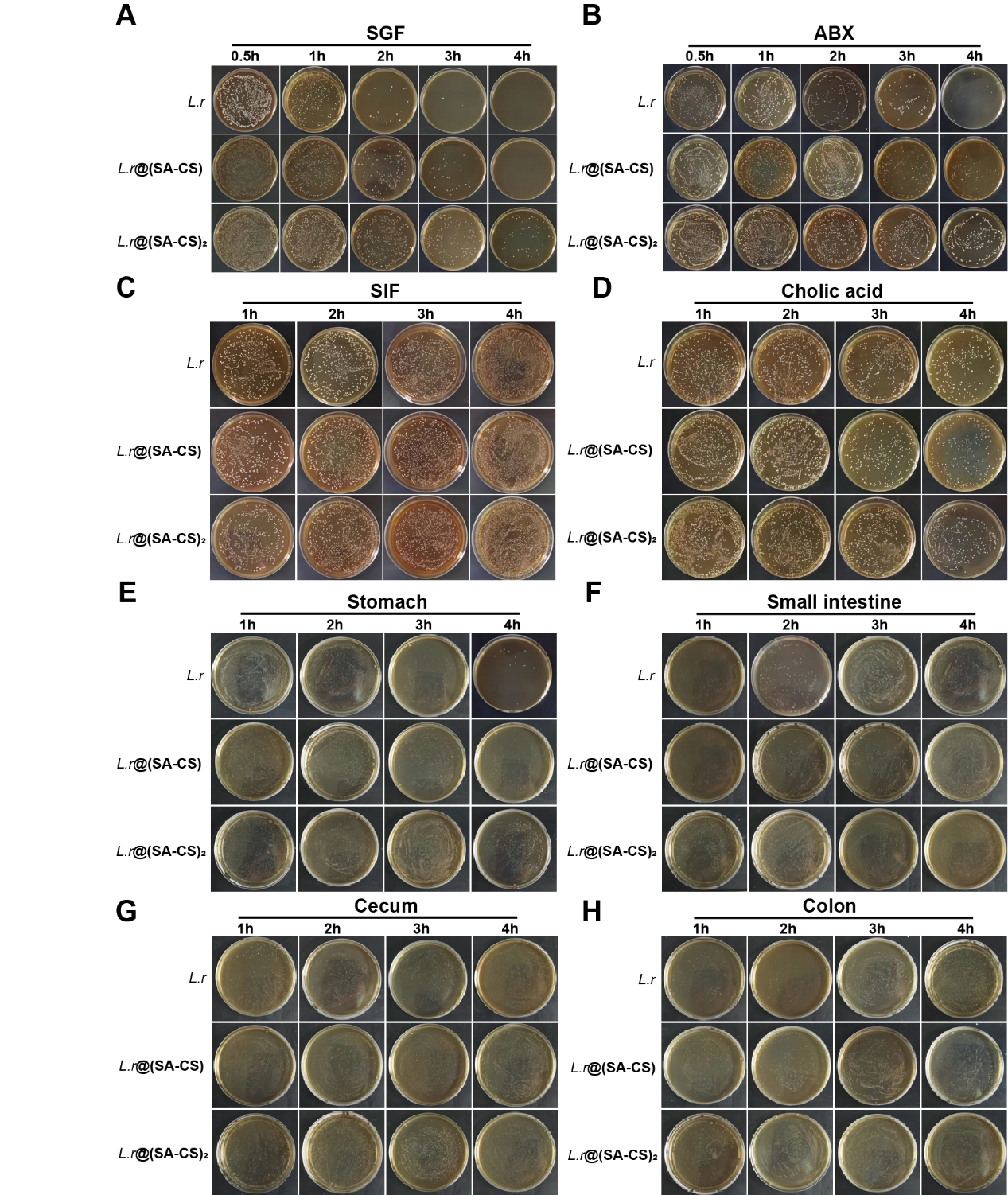


**Figure S2.** Representative photographs of solid MRS agar plates of *L.r*. (A to D) *L.r*, [*L.r*@(SA-CS](mailto:L.r@(SA-CS)), and *L.r*@(SA-CS)_2_ (100μL, *L.r*, 4.0×10^5^ CFUs) after treatment with (A) SGF, (B) ABX, (C) SIF, or (D) Cholic acid in vitro (n = 3). (E to H) *L.r* in the (E) stomach, (F) small intestine, (G) cecum, and (H) colon.at the indicated time points after gavage of 4.0 × 10^8^ CFUs of *L.r*, [*L.r*@(SA-CS](mailto:L.r@(SA-CS)) and *L.r*@(SA-CS)_2_ in vivo (n = 3).


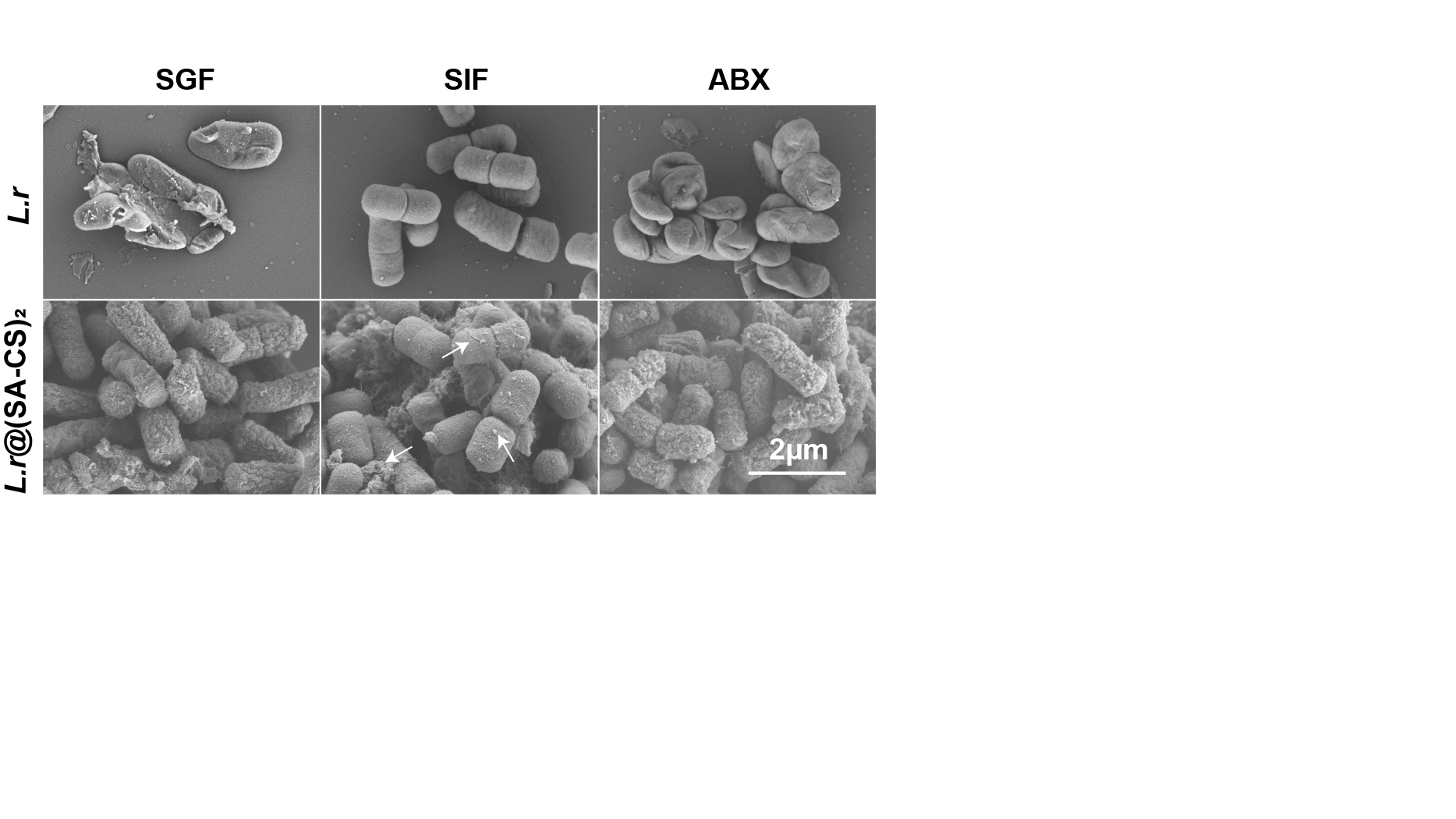


**Figure S3.** Typical SEM images of *L.r*, and *L.r*@(SA-CS)_2_ in SGF, SIF and ABX after 2 hours. The white arrow points to the cracked gel. Scale bar, 2 μm.


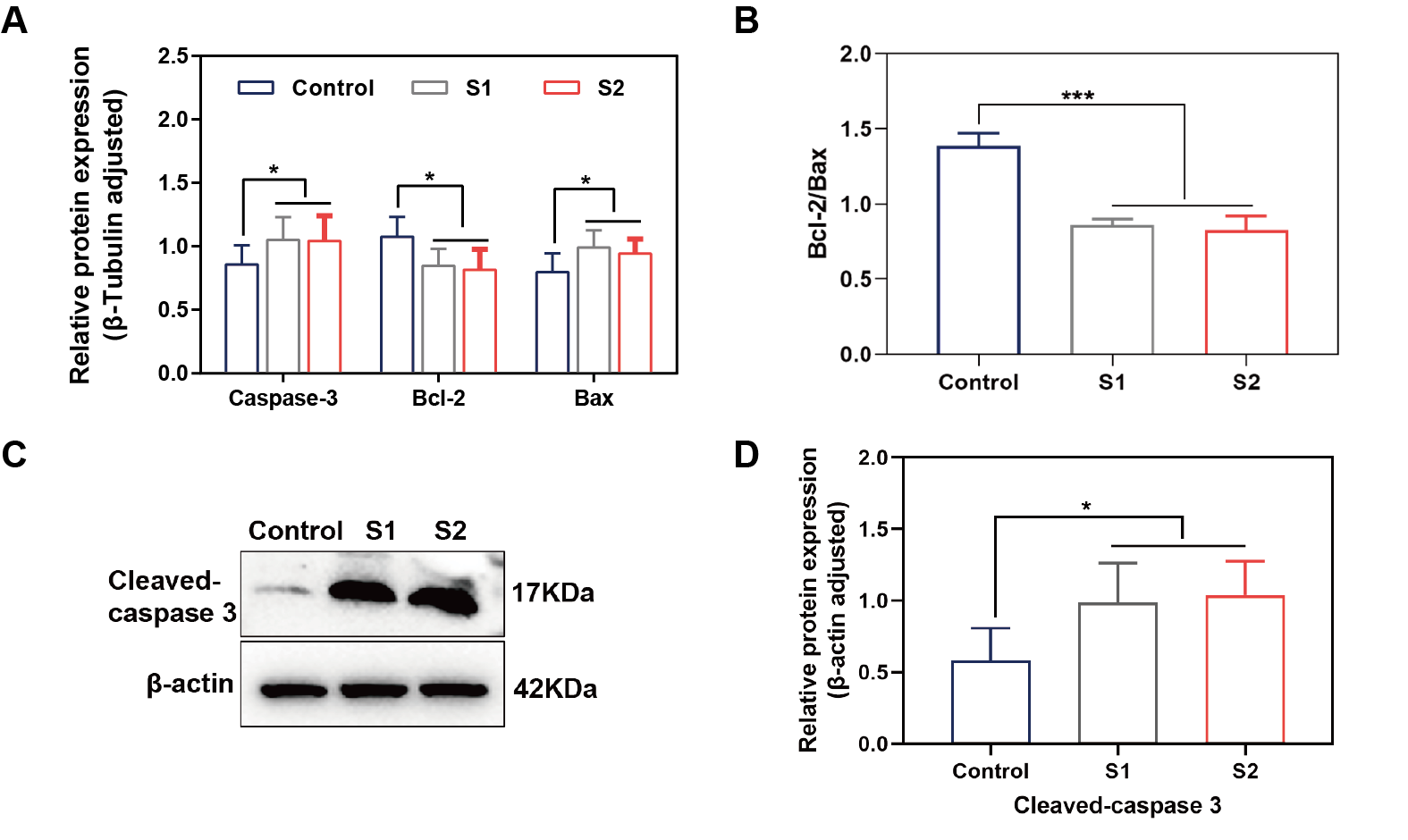


**Figure S4.** (A)Semi-quantitative analysis of apoptosis-related proteins (Caspase-3, Bcl-2, Bax) in Figure 3E. (B) Histogram of Bcl-2/Bax was used to detect the apoptosis of cells after co-incubation of supernatant and cells. (C) The levels of Cleaved-caspase 3 protein in CT26 tumor cells treated with S1 or S2, and the semi-quantitative analysis of Cleaved-caspase 3 protein (D). (S1: Supernatant collected after *L.r* was shaken at 37°C for 12 hours, S2: Supernatant collected after *L.r*@(SA-CS)_2_ was shaken at 37°C for 12 hours (n=3). *p < 0.05, ***p < 0.001.)


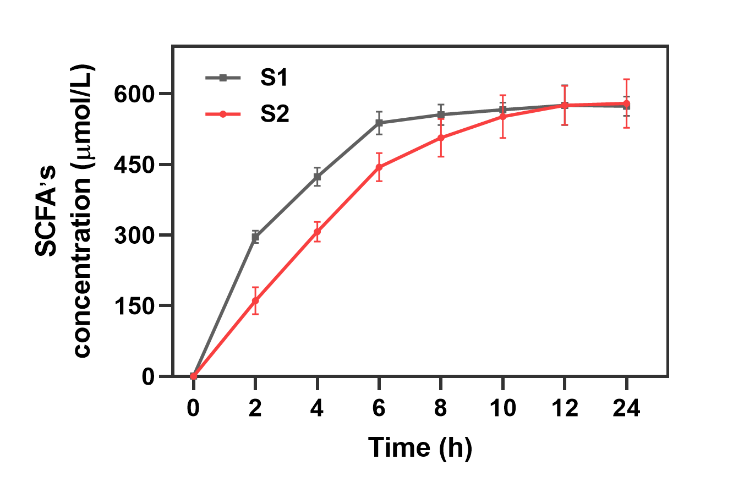


**Figure S5.** Release curve of SCFAs about *L.r* or *L.r*@(SA-CS)_2_ within 24h. (S1: Supernatant collected after *L.r* was shaken at 37°C for 12 hours, S2: Supernatant collected after *L.r*@(SA-CS)_2_ was shaken at 37°C for 12 hours. n=5).


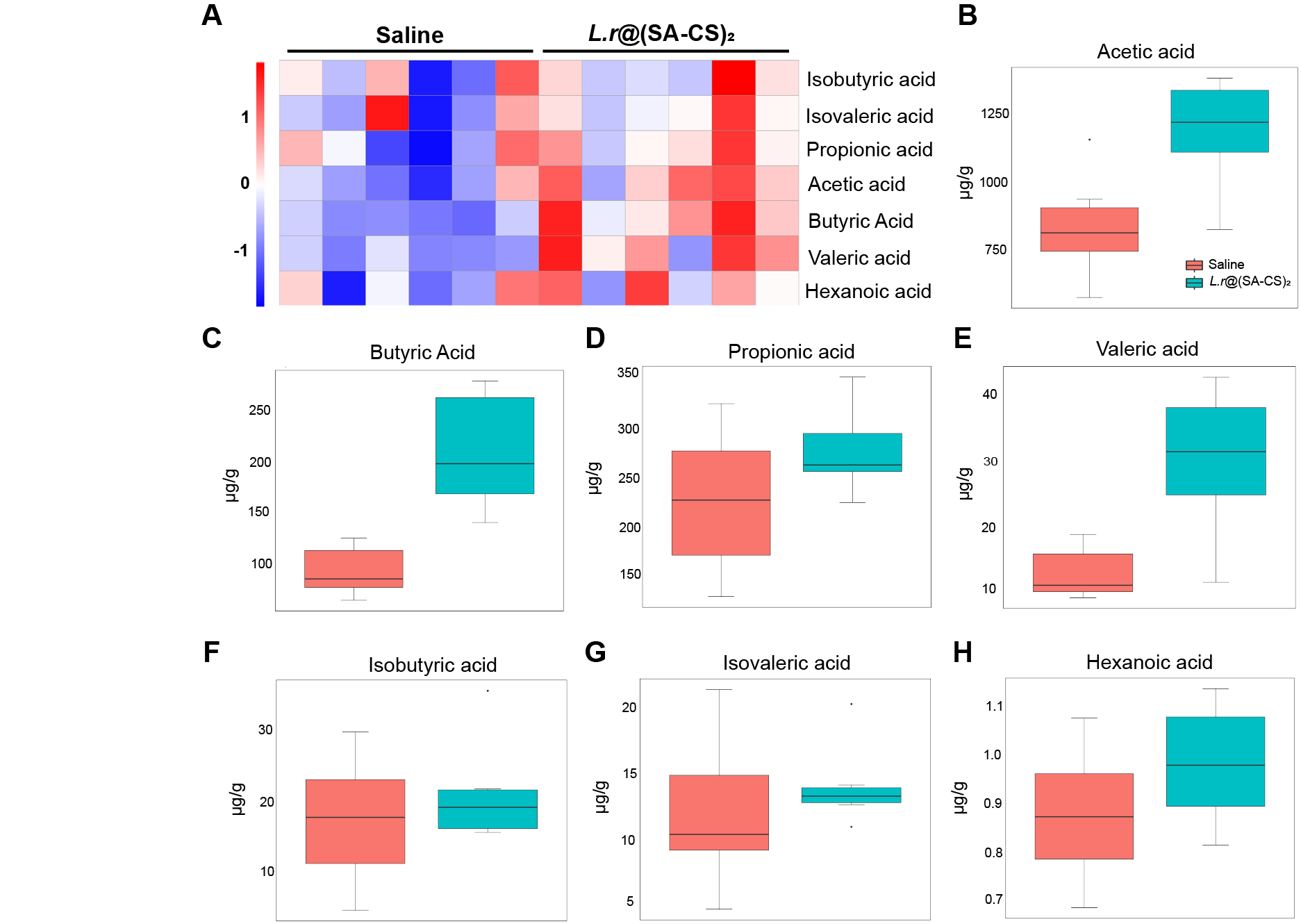


**Figure S6.** LC-MC analysis of SCFAs content in healthy mouse feces after gavage of Saline and [*L.r*@(SA-CS)_2_](mailto:L.r@(SA-CS)2) microgel respectively. (A) Heat map of the difference of SCFAs composition between the two groups. Comparison of specific contents of different metabolites, (B) Acetic acid, (C) Butyric acid, (D) Propionic acid, (E) Valeric acid, (F) Isobutyric acid, (G) Isovaleric acid, (H) Hexanoic acid in SCFAs between two groups. (n=6)


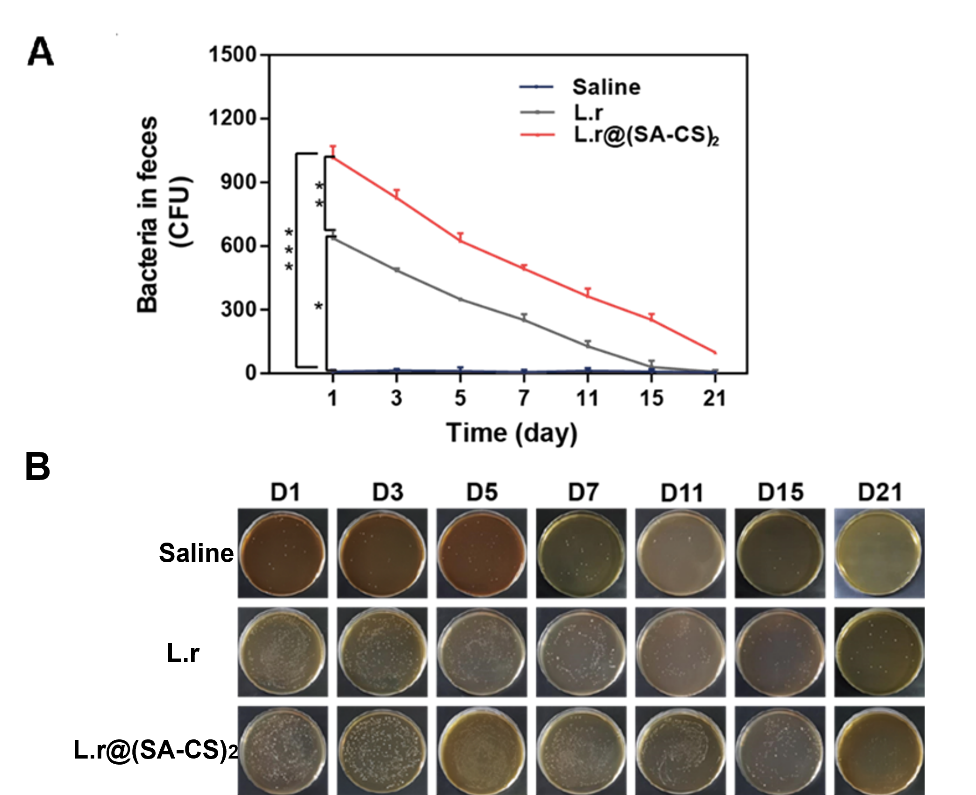


**Figure S7.** (A) An analysis of live *L.r*. in mouse feces at different stages of development. *p < 0.05, **p < 0.01, ***p < 0.001. (B) Representative photographs of solid MRS agar plates of *L.r*, gavaged with Saline, *L.r*, ad [*L.r*@(SA-CS)_2_](mailto:L.r@(SA-CS)2) (n = 3).


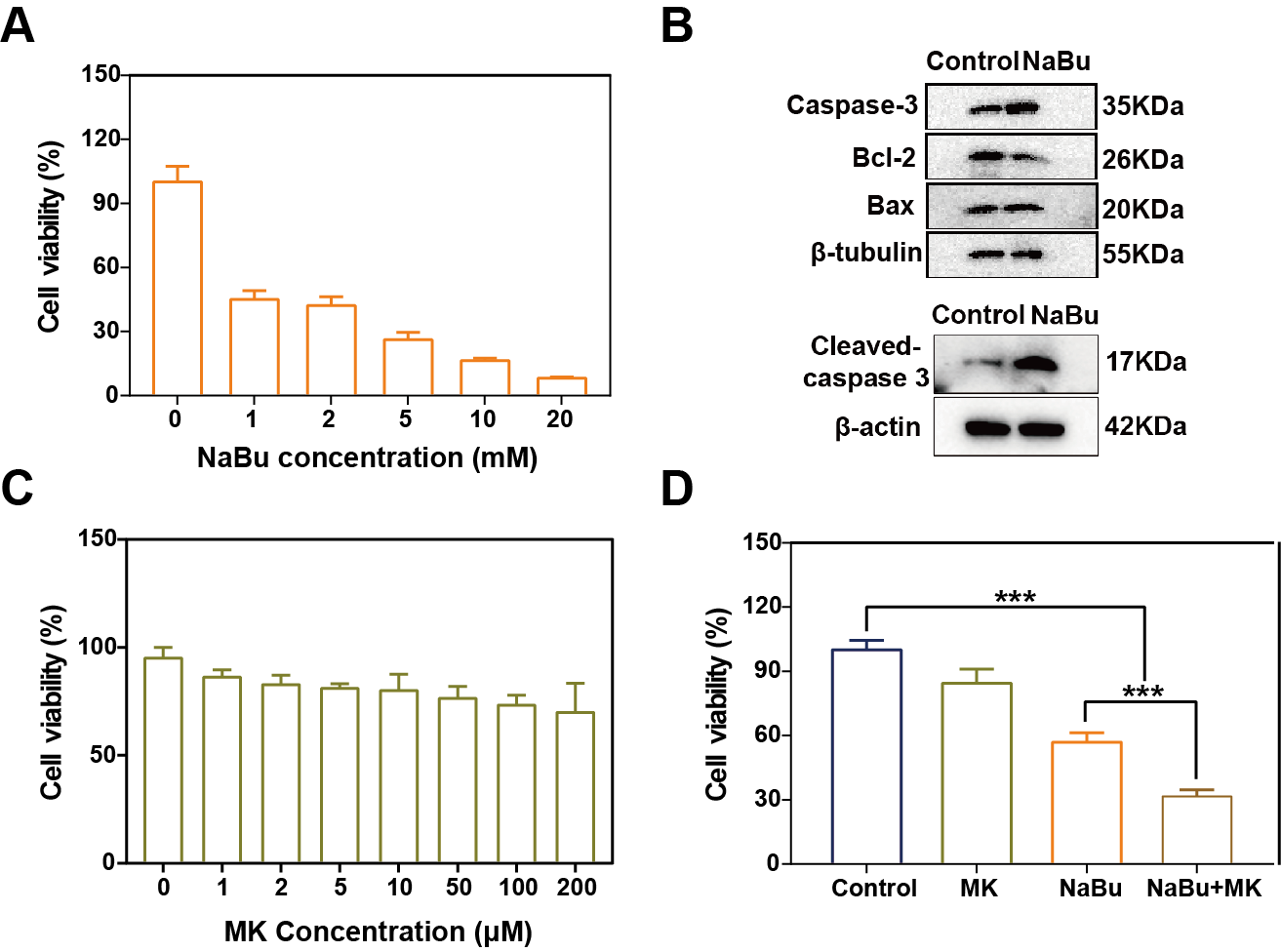


**Figure S8. Effects of sodium butyrate and MK on CT26 cells.** (A) A CCK8-based assay detected the killing effect of NaBu on CT26 cells. (B) NaBu induced the expression of apoptosis-related proteins (Caspase-3, Bcl-2, Bax, Cleaved-caspase 3) in tumor cells by western blotting (1.5mL, NaBu, 1mM) (n=3). (C) A cytotoxicity test was performed on MK using CCK8. (D) NaBu combined with MK killed cells when detected by CCK8 (1μM MK, 1mM NaBu, 1μM MK+1mM NaBu) (n=5).


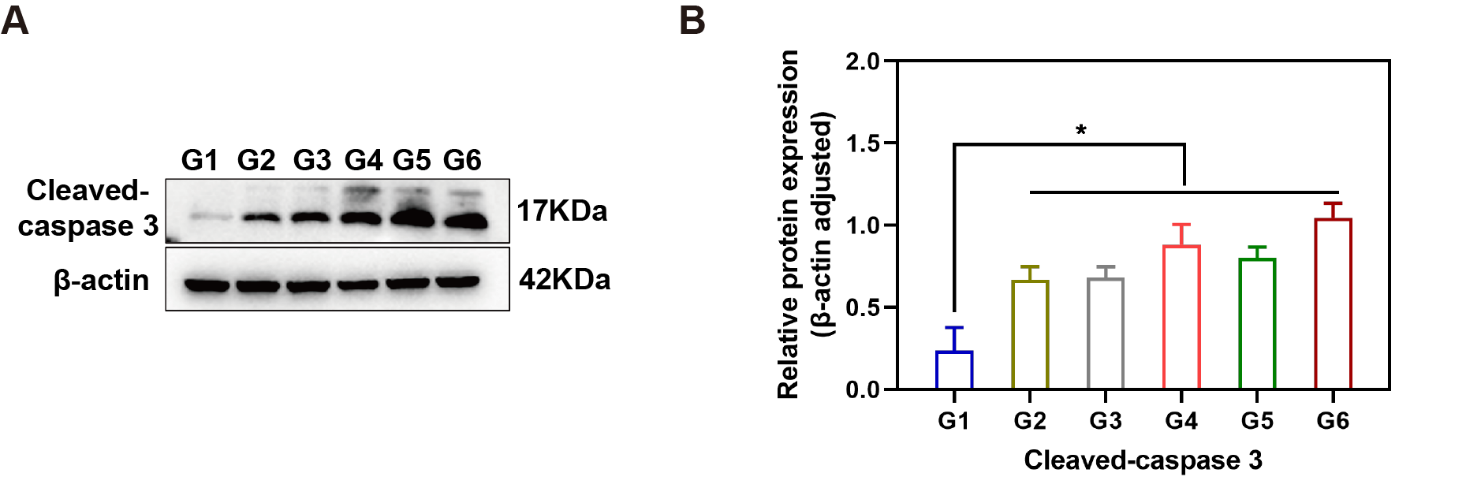


**Figure S9.** (A) The levels of Cleaved-caspase 3 protein in tumor tissues of each group were detected by WB, and the semi-quantitative analysis of Cleaved-caspase 3 protein (B). (n=3, *p < 0.05)
